# Engineering durable superhydrophobic layered double hydroxide coatings *via* incorporation of a nano-formulated quinoline-based ionic liquid for enhanced corrosion protection

**DOI:** 10.1039/d6ra05208h

**Published:** 2026-09-08

**Authors:** Eman M. Saadallah, Ahmed R. Rabee, Essam M. Sharshira, Mohamed Hagar, Emad Matter, Azza Shaker, Mohamed Elshahat Mohamed

**Affiliations:** a Chemistry Department, Faculty of Science, Alexandria University P.O. Box 426 Alexandria 21321 Egypt dressamsharshira@yahoo.com mohamed.hagar@alexu.edu.eg elshahatchemist93@gmail.com; b Chemistry Department, Faculty of Science, Damanhour University Damanhour 22511 Egypt

## Abstract

In this study, a newly nano-formulated quinoline-based ionic liquid (NILq) was successfully synthesized and characterized. NILq was incorporated into a Ni–Al layered double hydroxide (LDH) matrix to fabricate a superhydrophobic coating, LDH–NILq–SA, on carbon steel *via* one-step electrodeposition to increase its corrosion resistance in marine and industrial infrastructure applications. The synthesized ionic liquid was characterized by FTIR, ^1^H NMR, ^13^C NMR and HRMS spectroscopy. The NILq exhibited a spherical morphology observed by TEM and a highly positive surface charge of +27.8 mV from zeta potential measurements, indicating excellent colloidal stability. The incorporation of NILq modulated the LDH growth behavior, generating a hierarchical nano-architecture capable of stabilizing the Cassie–Baxter wetting state and enhancing long-term interfacial durability. The optimized LDH–NILq–SA coating demonstrated exceptional superhydrophobicity with a sliding angle and water contact angle of 0.5°, 166.5° respectively. Surface investigation by SEM and AFM revealed a well-developed layered structure characteristic of layered double hydroxides with optimized surface roughness (*R*_a_ = 369.9 nm). The coating exhibited prominent chemical stability, maintaining superhydrophobicity over pH range (1–13), and remarkable mechanical durability, withstanding 1050 mm of abrasion under 5 kPa load. The optimized coating exhibited exceptional thermal stability, maintaining superhydrophobicity up to 200 °C with full regenerability after thermal degradation. Electrochemical measurements performed in 0.5 M NaCl solution demonstrated that the 30 ppm LDH–NILq–SA coating achieved a protection efficiency of 99.5%, significantly outperforming the NILq-free LDH–SA coating. This work presents an effective strategy for fabricating durable, high-performance superhydrophobic coatings with promising applications in corrosion protection for marine and industrial infrastructure.

## Abstract 2

Synthesis and characterization of new nano-formulated quinoline-based ionic liquid and incorporated it with layered double hydroxide to enhance the durability of superhydrophobic coating.

## Graphical abstract

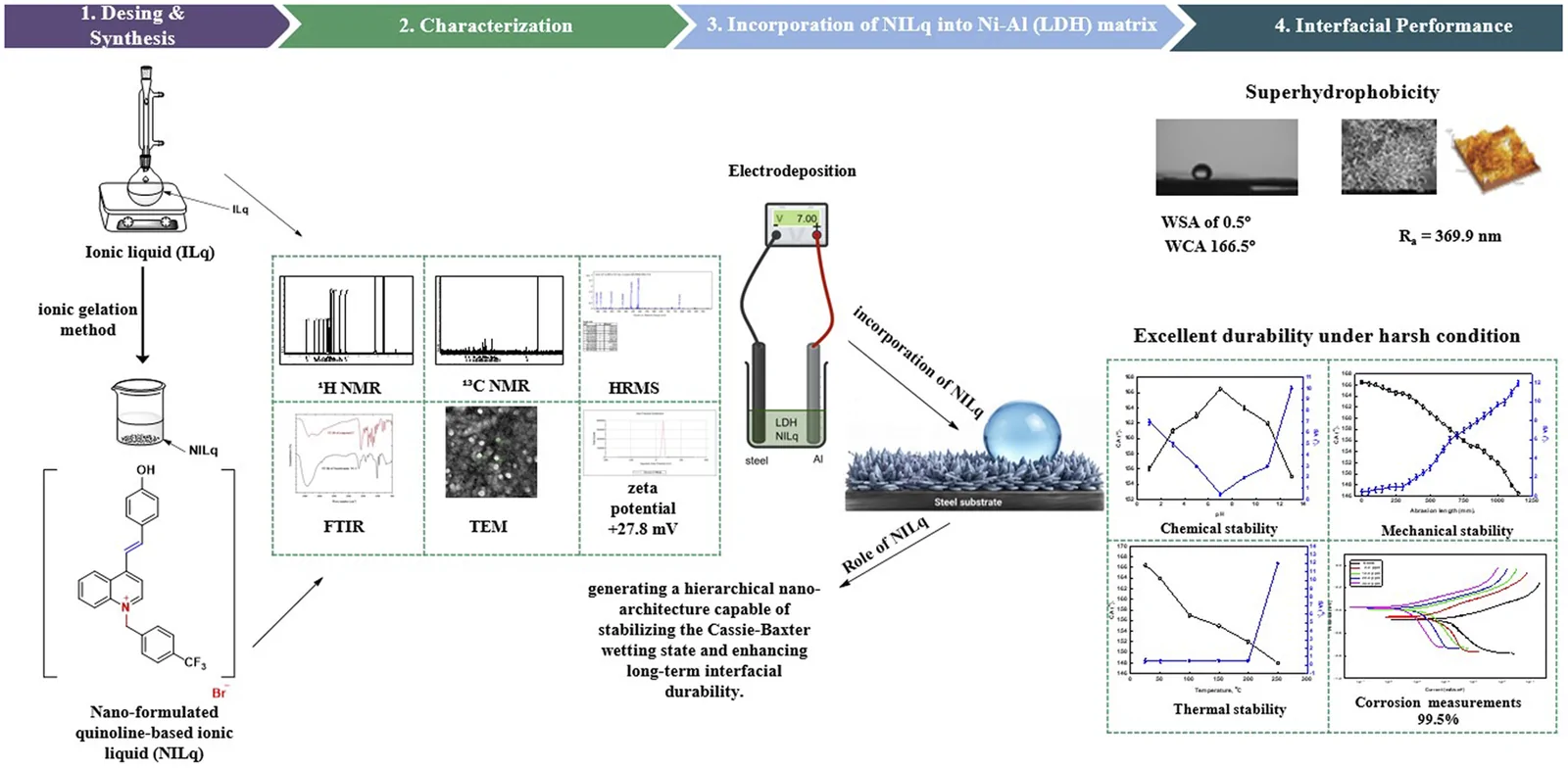

## 1. Introduction

Corrosion is widely regarded as a detrimental phenomenon that poses a significant threat to the long-term sustainability and economic viability of industrial infrastructure, leading to substantial financial losses worldwide.^1–5^ Carbon steel is one of the most important construction materials as it is widely used in marine and industrial fields. That is due to its low cost and favorable physical and chemical properties. However, corrosion failures induced by salinity and moisture remain serious and costly challenges during application.^6–8^

There are a lot of protective methods that have been developed to inhibit corrosion toward carbon steel including, sacrificial anode,^9^ corrosion inhibitors,^10^ organic coatings^11^*etc.* While effective, each method has inherent limitations.

A promising alternative is the development of superhydrophobic (SHP) coatings, inspired by the Lotus leaf and characterized by a water contact angle higher than 150° and a sliding angle lower than 10.^12–15^ It is considered as one of the most ideal strategies towards corrosion protection of the metals and alloys primarily due to their effective barrier effect. The unique wettability characteristics inhibit the accumulation of aggressive corrosive ions and pollutants on the surface, thereby enhancing corrosion resistance.^16–19^ The development of SHP surface relies on two primary parameters: the creation of hierarchical micro–nano roughness on the solid substrate and the presence of low surface energy. Nanostructures represent an attractive approach for generating nano-scale surface roughness through their deposition onto metallic solid substrates.^20^ Surface energy can be effectively lowered through chemical modification by incorporating low surface energy materials such as stearic acid, fluorosilanes, and lauric acid.^21^ One of the most effective surface modifiers is stearic acid, owing to its long hydrocarbon chain containing methyl and methylene groups, which can significantly reduce the surface free energy of materials. Moreover, stearic acid exhibits good biocompatibility and lower toxicity compared to many synthetic polymers, as it is a naturally occurring fatty acid and a major component of fats.^22,23^

Layered double hydroxides (LDHs) are a class of two-dimensional materials characterized by the general formula, [M_(1−*x*)_^2+^ M_(1−*x*)_^3+^(OH)_2_]^*x*^ + (A^*n*−^)_*x*/*n*_·*z*H_2_O. In this structure, M^2+^ and M^3+^ represent divalent and trivalent metal cations, respectively, while A^*n*−^ denotes interlayer anions such as carbonate, nitrate, chloride, or bromide.^24,25^

LDHs can be employed to construct rough surface architecture, and when modified with low-surface-energy materials, they enable the fabrication of SHP coats. In this system, LDHs not only provide the required hierarchical roughness but also contribute to corrosion protection by adsorbing aggressive anions.^26^ LDHs can significantly enhance the adhesion strength between the coating and the substrate. Based on this concept, integrating LDHs with SHP coatings represents a viable strategy for improving overall corrosion resistance.^26^

Ionic liquids (ILqs) are molten salts that are composed of an organic cation that are large asymmetric structure and organic or inorganic anions that are much smaller in size than the cations.^27^ The organic cation mostly contains phosphorus or nitrogen atoms such as, imidazolium,^28^ triazolium,^29^ pyridinium,^30^ pyrazolium.^31^ Due to their irregular structures, the charges are delocalized.^27^ However, current investigations indicate that ionic liquids derived from imidazolium, pyridinium, and pyrazolium cations exhibit pronounced therapeutic efficacy, largely attributable to their elevated charge density and hydrophobic character.^32^ Moreover, the chemical inertness of imidazole and together with toxicity associated with pyridine limit the further development and diversification of ionic liquids. In response to these limitations, quinoline characterized by its relatively low toxicity, has been selected as an alternative cationic component.^32,33^ Ionic liquids have a unique property such as non-flammability, low vapor pressure, high thermal and chemical stability, and good electrical conductivity.^34–36^ As a result of these properties, ionic liquids have been used in a lot of fields such as electrochemistry, nanotechnology, analytical chemistry, extraction processes, and many more.^37–39^ Ionic liquids have been widely employed as functional additives to enhance the quality and performance of a broad range of materials, including lubricants, paints, shampoos, conditioners, fabric softeners, adhesives, and detergents.^36^

The integration of a nano-formulated quinoline-based ionic liquid with LDH for the fabrication of multifunctional superhydrophobic coatings has not been previously reported. In this work, a novel NILq was synthesized and employed as a functional additive in Ni–Al LDH coatings to develop a robust superhydrophobic system on carbon steel. The incorporation of NILq is expected to modulate the nucleation and growth behavior during electrodeposition, leading to improved surface morphology and enhanced physicochemical properties. The resulting coatings were systematically investigated in terms of wettability, corrosion resistance, and thermal, mechanical, and chemical stability. Furthermore, the structure property performance relationship was explored to elucidate the role of NILq in enhancing the overall coating performance. This study provides new insights into the design of advanced hybrid coatings with improved durability and multifunctionality for practical corrosion protection applications.

## 2. Material and method

### 2.1. Material and equipment

In this study, a sheet of carbon steel (20 × 50 × 2 mm^3^) and aluminum (20 × 50 × 2 mm^3^) were used as substrates. All materials and equipment are included in SI.

### 2.2. Synthesis of (*E*)-4-[2-(4-hydroxyphenyl)ethenyl]quinoline (3)

The compound was previously prepared following the reported procedure with some modifications.^40^ As a mixture of *p*-hydroxybenzaldehyde (0.2 g, 1.63 mmol) and 0.5 mL of acetic anhydride was refluxed for 6 h. Subsequently, 4-methylquinoline (0.28 g, 1.96 mmol) was introduced, and the reaction mixture was further refluxed for 24 h. Thereafter, ethanolic solution (15 mL) of potassium hydroxide (0.18 g, 3.27 mmol) was added, and reflux continued for an additional 24 h. Upon completion of the reaction, the mixture was poured into ice water, and the resulting precipitate was collected by Büchner filtration and recrystallized from absolute ethanol to afford a desired product as yellow precipitate; IR(KBr) *ν*_max_ (cm^−1^): 3395 (OH), 3067 (=CH, sp^2^ stretch), 1575 (HC=CH), were observed as strong bands.

### 2.3. Synthesis of (*E*)-4-(4-hydroxystyryl)-1-(4-(trifluoromethyl)benzyl)quinolinium bromide (5)

A mixture of 4-[2-(4-hydroxyphenyl)ethenyl]quinoline (3) (0.150 g, 0.60 mmol) and 4-(trifluoromethyl)benzyl bromide (4) (0.145 g, 0.60 mmol) was refluxed in 15 mL of acetonitrile for 24 h. The reaction progress was checked by thin-layer chromatography (TLC) using mixed eluent (ethyl acetate: methanol, 3 : 0.5) till completion. After cooling to room temperature, the excess solvent was allowed to evaporate, and the resulting product was recrystallized from absolute ethanol to afford the target ionic liquid (compound 5) as orange crystals; IR (KBr) *ν*_max_ (cm^−1^): 3373 (OH), 3075 (=CH, sp^2^ stretch), 1580 (HC=CH) were observed as strong bands; ^1^H NMR (500 MHz, DMSO-d6) *δ*_H_: 10.31 (s, 1H, **OH**), 9.47 (d, *J* = 6.5 Hz, 1H, Ar–H), 9.05 (d, *J* = 3.0 Hz, 1H, Ar–H), 8.55 (d, *J* = 6.0 Hz, 1H, Ar–H), 8.25 (m, 1H, Ar–H), 8.211 (d, *J* = 9.0 Hz, 1H, Ar–H), 8.18 (d, *J* = 9.5 Hz, 1H, Ar–H), 8.11 (m, 1H, Ar–H), 7.94 (2d, *J* = 7,8.5 Hz, 1H, Ar–H), 7.88 (d, *J* = 8.5 Hz, 2H, Ar–H), 7.72 (d, *J* = 8.5 Hz, 2H, Ar–H), 7.50 (d, *J* = 8.5 Hz, 2H, Ar–H), 6.89 (d, *J* = 8.5 Hz, 2H, **HC=CH**), 6.31 (s, 2H, **CH**_**2**_); ^13^C NMR (125 MHz, DMSO-d6) *δ*_C_: 161.2, 154.9, 148.4, 145.3, 138.4, 136.3, 135.9, 132.0, 129.7, 128.2, 127.3, 127.2, 126.5, 126.4, 119.7, 116.7, 116.6, 116.5, 116.2 (Ar–C), 113.1 (HC=CH), 58.9 (CH_2_); HRMS (ESI) *m*/*z* calcd for C_25_H_20_NOBrF_3_ [M + 1]^+^: 486.068; found 486.048.

### 2.4. Synthesis of nano-formulated quinoline based ionic liquid (NILq)

The chitosan nano-formulation was synthesized *via* the ionic gelation method.^41^ Briefly, 0.57 g of chitosan was dissolved in 75 mL of 2% (v/v) acetic acid and stirred continuously for 1 hour to ensure complete solubilization. Subsequently, 0.045 g of an ionic liquid, pre-dissolved in 5 mL of dimethyl sulfoxide (DMSO), was added dropwise under controlled conditions, and the mixture was stirred for an additional 1 h to promote uniform interaction. Thereafter, 0.1875 g of anhydrous sodium tripolyphosphate (TPP), dissolved in 54 mL of distilled water, was introduced dropwise into the reaction mixture. The reaction mixture was allowed to stir overnight to facilitate ionic crosslinking and nanoparticle formation. The resulting nano-formulated compound was characterized and confirmed using infrared (IR) spectroscopy and transmission electron microscopy (TEM).

### 2.5. Synthesis of Ni–Al LDH superhydrophobic coating on steel substrates

Carbon steel and aluminum sheets (20 mm × 50 mm × 2 mm) were mechanically abraded by abrasive papers with a grade size of 120, followed by rinsing with double-distilled water. The insulation of steel substrate was performed by using epoxy resin, and a well-defined surface area of 2 cm^2^ was left exposed to the electrodeposition bath. Prior to electrodeposition, the exposed working area was further mechanically abraded using abrasive papers with grade sizes of 120, 320, and 600, and subsequently rinsed with double-distilled water.

Ni–Al LDH-coated carbon steel was fabricated using one-step process in a two-electrode system. The carbon steel substrate acted as the cathode, whereas an aluminum plate of identical size functioned as an anode. Both electrodes were arranged in a parallel configuration, maintaining a fixed interelectrode spacing of 2.0 cm.

The preparation of bath solution was employed by solubilizing 1.0 M nickel chloride and 1 M nickel sulfate in double-distilled water at a 1 : 4 molar ratio, respectively. The pH of the solution was maintained at 6.70 by using NaOH (pH 11.5). Electrodeposition was performed under potentiostatic conditions at an applied voltage of 7.0 V for 6.0 minutes (optimum conditions).

The electrodeposition was performed in the absence and presence of different concentrations of nano-formulated ionic liquid (10, 20, and 30 ppm). After electrodeposition, to remove remaining salt residues the coated steels were thoroughly washed with distilled water then allowed to dry at room temperature for 24 h. Superhydrophilicity was achieved by immersing the LDH-coated samples in a 0.01 M ethanolic stearic acid solution for 15 min, followed by drying at room temperature for 24 h. The obtained superhydrophobic samples on the steel substrate were donated as LDH–SA, in the absence of nano-formulated ionic liquid, and LDH–NILq–SA, in the presence of nano-formulated ionic liquid. Then, the prepared samples were subjected to different characterization and evaluation techniques.

### 2.6. Electrochemical measurements

All electrochemical measurements were carried out using a conventional three-electrode cell, comprising the superhydrophobic coated steel sample as the working electrode, a graphite as a counter electrode, and an Ag/AgCl/KCl_(sat.)_ as a reference electrode. Under ambient conditions the tests were conducted in a 3.5 wt% NaCl solution. To steady the rest potential before tests, both bare and superhydrophobic coated steel specimens were introduced into the solution for twenty minutes. Electrochemical impedance spectroscopy (EIS) was then performed over a frequency range of 100 kHz to 0.01 Hz with a sinusoidal perturbation amplitude of 10 mV around the rest potential. The PDP measurements were performed within a potential range of ±300 mV at a scan rate of 30 mV min^−1^. All experiments were repeated to ensure reproducibility, with results within ±2% error margin.

### 2.7. Chemical and mechanical stability

The chemical stability of the steel coated by LDH–SA and the optimum LDH–NILq–SA (30 ppm of the nano-formulated ionic liquid) was evaluated by immersing different samples in solutions of varying pH (1–13) for 30 min at room temperature. The pH of the solutions was adjusted using 0.5 M H_2_SO_4_ and 0.5 M NaOH. After immersion, the samples were dried and the WCA and SA were measured for each sample.

The mechanical stability of the superhydrophobic LDH samples without and with the optimal nano-formulated ionic liquid concentration was investigated *via* scratch tests. The samples were mechanically abraded with 600-grit SiC paper under an applied pressure of 5 kPa. WCA and SA measurements were performed after every 50 mm of abrasion to assess the durability of the coatings. The reported chemical and mechanical abrasion resistance data represent the average of three tests.

## 3. Results and discussion

### 3.1. Chemistry

Our work was focused on the design and synthesis of newly quinoline based ionic liquid for targeting superhydrophobic coating as a new technique for corrosion protection. As adopted in Scheme 1, the aldol-condensation of *p*-hydroxybenzaldehyde (1) and 4-methyl quinoline (2) to afford compound 4-[2-(4-hydroxyphenyl)ethenyl]quinoline (3) was carried out as reported in previous work,^40^ with some modifications as mentioned in methodology in Section 2.2. Moreover, the targeted ionic liquids (*E*)-4-(4-hydroxystyryl)-1-(4-(trifluoromethyl)benzyl)quinolinium bromide (5) was prepared by reflux the mixture of 4-[2-(4-hydroxyphenyl)ethenyl]quinoline (3) and 4-(trifluoromethyl)benzyl bromide (4) in 15 mL acetonitrile for 24 h to afford the targeted ionic liquid (5). The structures of the synthesized compound 5 were validated *via* spectroscopic data. The proton nuclear magnetic resonance (^1^H NMR) spectrum demonstrated specific peaks for phenolic OH, HC=CH and CH_2_ at 10.31, 6.89 and 6.31 ppm; respectively. Its ^13^C NMR spectrum showed peaks at *δ*_C_: 113.1 and 58.9 corresponds to HC=CH and CH_2._ Moreover, its IR spectrum showed broad band of OH at 3373 cm^−1^, and sharp peak of HC=CH was observed at 1580 cm^−1^. All spectroscopic data are included in SI.

**Scheme 1:**
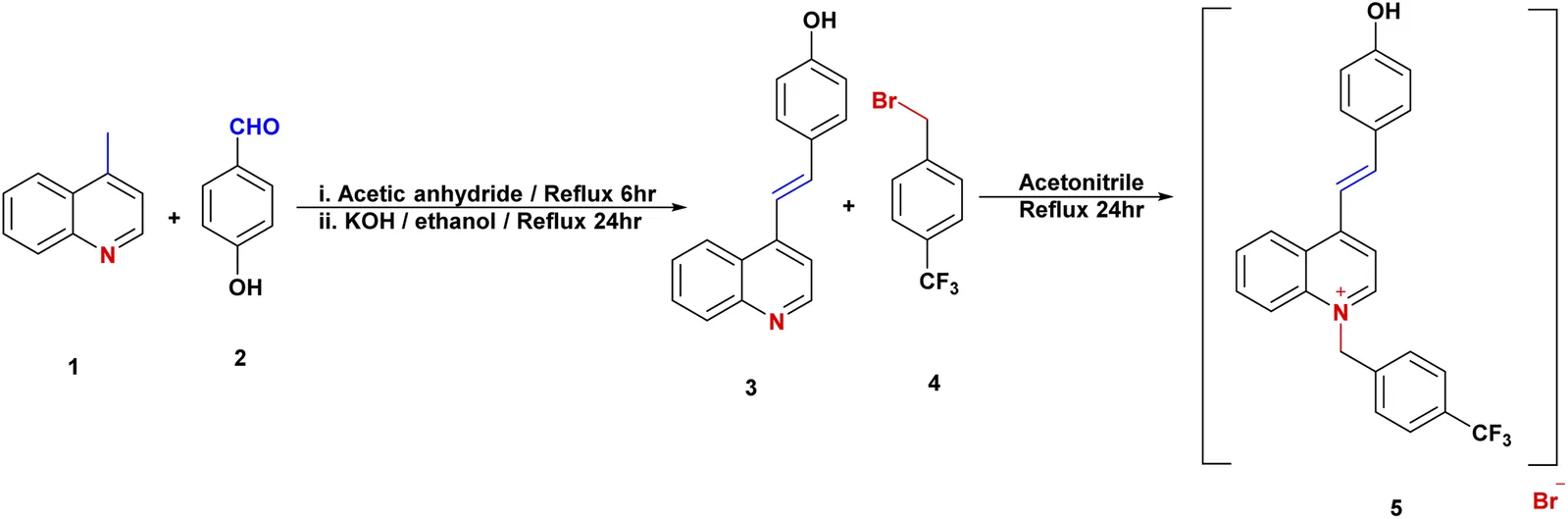
Schematic methodology for the synthesis of quinoline based ionic liquid compound 5.

### 3.2. Chitosan nano-formulation for the synthesized ionic liquids

To confirm proper preparation of the synthesized nano formula, comprehensive characterization of the prepared nano-formulated ionic liquid NILq was performed using multiple analytical techniques. As shown in Fig. 1, the TEM images revealed a spherical particle in nano scale size range around 25–35 nm and confirm proper encapsulation. Nano size plays an important role in forming roughness on the surface of coat to make it superhydrophobic. The size range of 28 nm with ±5 nm that gives a great result on making surface rough as will show in AFM.

**Fig. 1:**
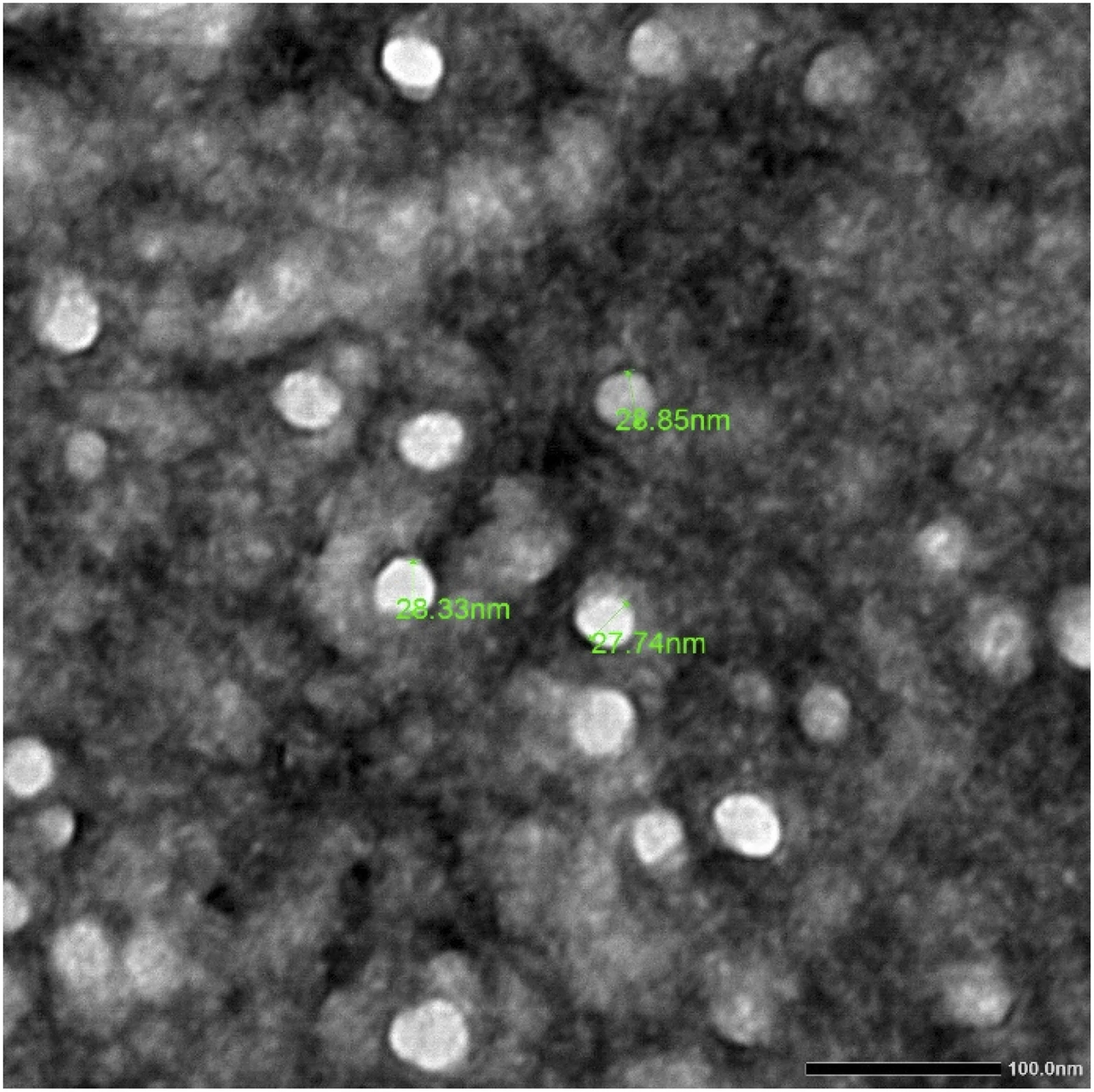
TEM images of NILq compound 5.

Moreover, the prepared nano formula NILq presented highly positively charged with zeta potential (*ζ*) up to +27.8 mV, indicating that the synthesized NILq displayed increased dynamic stability as shown in Fig. 2.

**Fig. 2:**
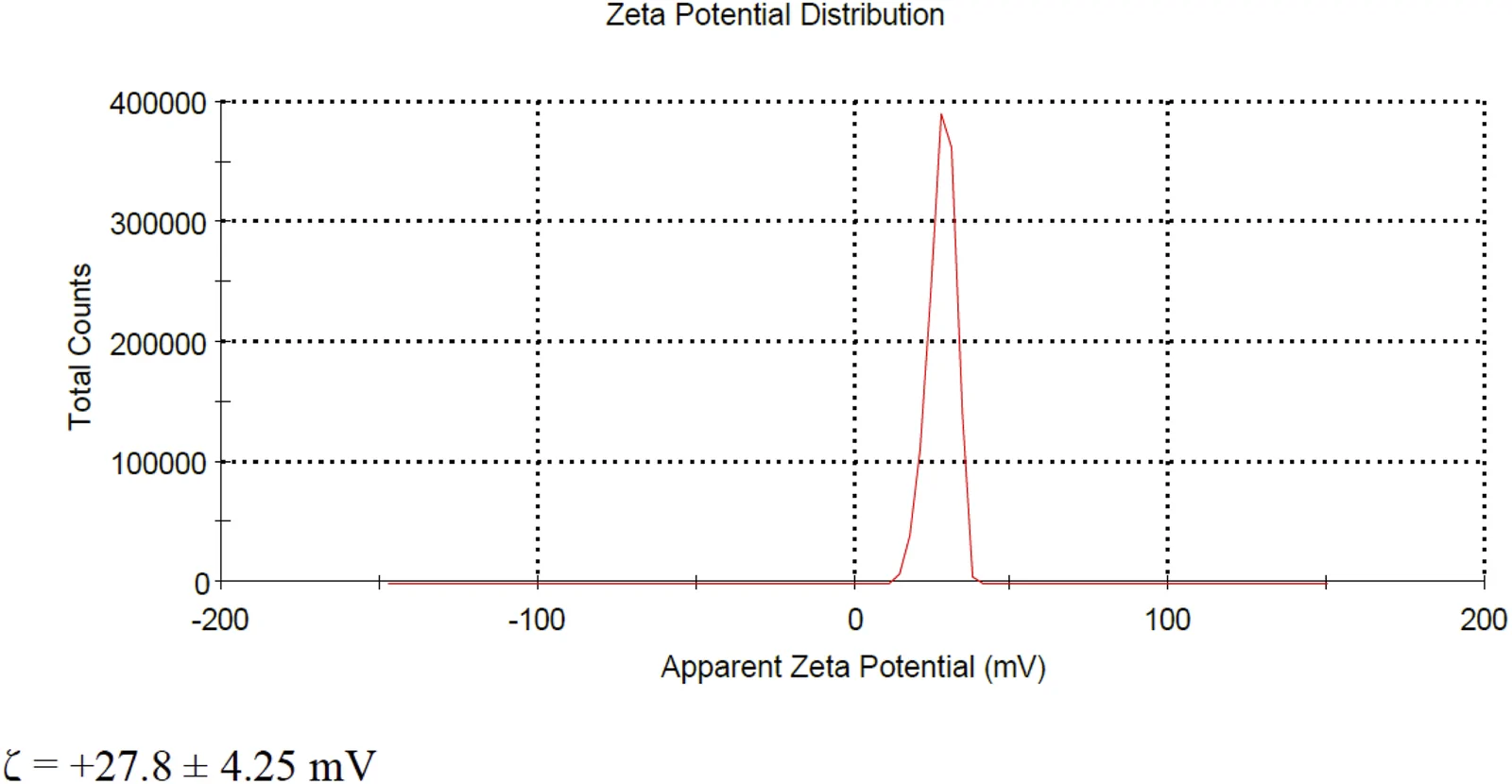
Dynamic light scattering (DLS) study of the synthesized nanoparticles NILq showing zeta potential distribution curve.

The FTIR spectrum for the synthesized compound 5 and its nano formula NILq is shown in Fig. 3. The nano-formulation showed broad bands of chitosan about 3400–2980 cm^−1^, indicating enhanced hydrogen bonding within the chitosan nano-matrix. Moreover, the aromatic C=C and phenolic C–OH bands show slight changes in position and intensity, suggesting intermolecular interactions between compound 5 and the nanocarrier. These spectral modifications confirm successful incorporation of compound 5 into the nano-formulation without structural degradation of its key functional groups.

**Fig. 3:**
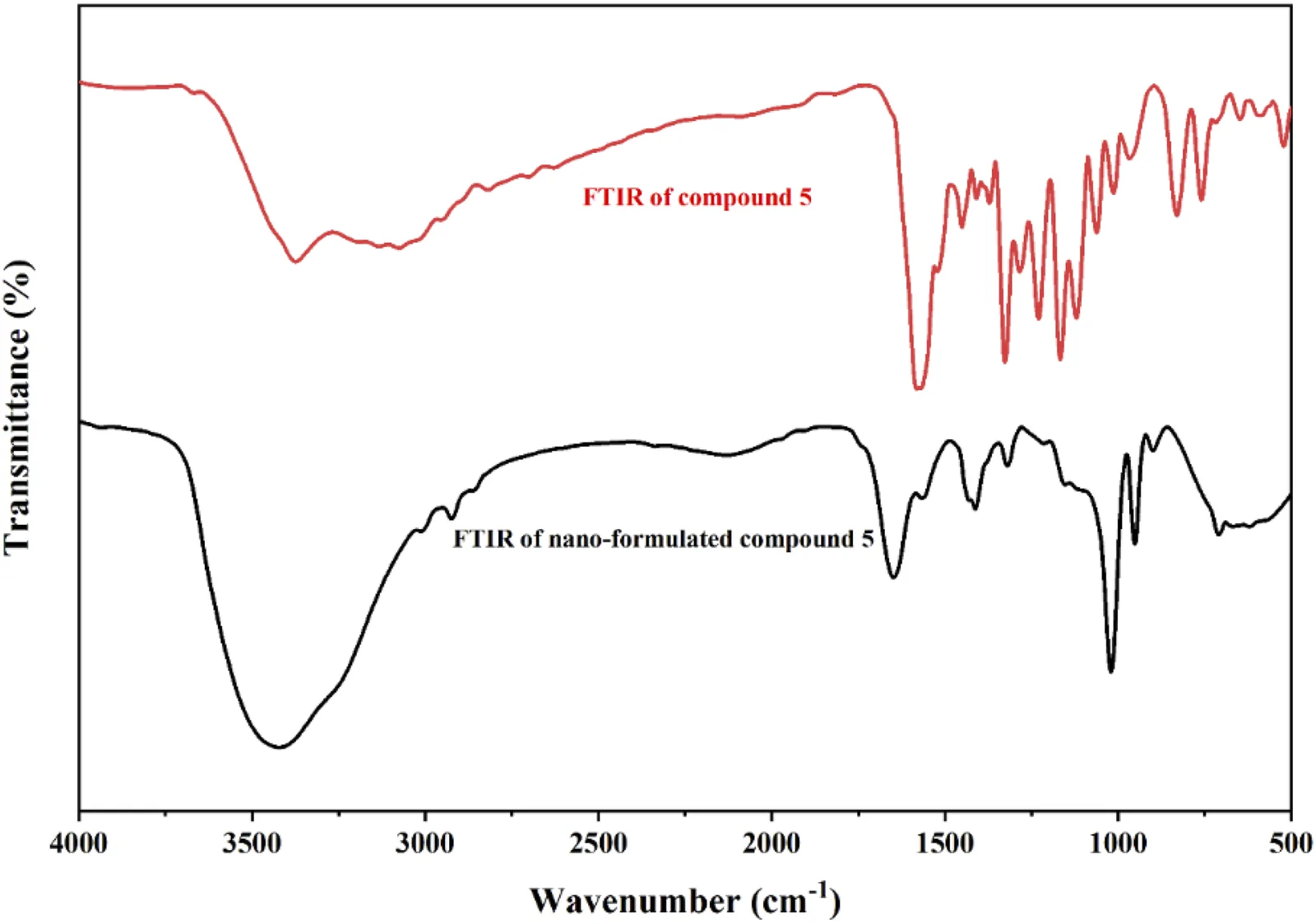
The FTIR spectra of compound 5 and its nano-formulated form NILq.

### 3.3. Formation of the Ni–Al LDH

The steel substrate was grafted by the Ni–Al LDH coating using a one-step cathodic electrodeposition method. The electrolyte, a modified Watts bath, consists of nickel sulfate and nickel chloride as the sources of divalent nickel ions (Ni^2+^).

This electrodeposition strategy relies on the *in situ* generation of trivalent aluminum ions (Al^3+^) from a sacrificial aluminum anode. During the process, the aluminum anode undergoes oxidation, providing the essential Al^3+^ cations for LDH formation:

Anodic reaction:

1 Al → Al^3+^ + 3e^−^

Simultaneously, at the steel cathode, the reduction of water molecules occurs. This cathodic reaction consumes electrons and generates hydroxide ions (OH^−^), leading to a progressive increase in the local pH at the electrode surface:

Cathodic reaction:

2 2H_2_O + 2e^−^ → H_2_↑ + 2OH^−^

Under the influence of the applied electric field, the Ni^2+^ ions from the electrolyte and the electrochemically generated Al^3+^ ions migrate toward the cathode. Upon reaching the alkaline diffusion layer at the cathode surface, these metal cations co-precipitate as a mixed hydroxide, forming a compact and adherent Ni–Al LDH film. The overall co-precipitation process can be represented as follows:

LDH formation:

3 (1 − *x*)Ni^2+^ + *x*Al^3+^ + 2OH^−^ + *n*H_2_O → [Ni_1−*x*_ Al_*x*_ (OH)_2_] (A^*n*−^)_*x*/*n*_·*n*H_2_O

where A^*n*−^ represents the interlayer anions (*e.g.* SO_4_^2−^, Cl^−^, OH^−^, and potentially CO_3_^2−^ from atmospheric CO_2_) incorporated from the electrolyte to balance the positive charge of the brucite-like layers.

This synergistic electrochemical–chemical mechanism facilitates the controlled one-pot growth of a uniform Ni–Al LDH coating on the carbon steel plate, providing the necessary hierarchical roughness for subsequent superhydrophobic modification.

### 3.4. Analysis of LDH–NILq interaction

To investigate the nature of the interaction between the NILq and the Ni–Al LDH framework, FTIR analysis was performed on the LDH–NILq composite. The spectrum is presented in Fig. 4 and compared with that of the free NILq (Fig. 3) to identify spectral changes indicative of chemical interactions.

**Fig. 4:**
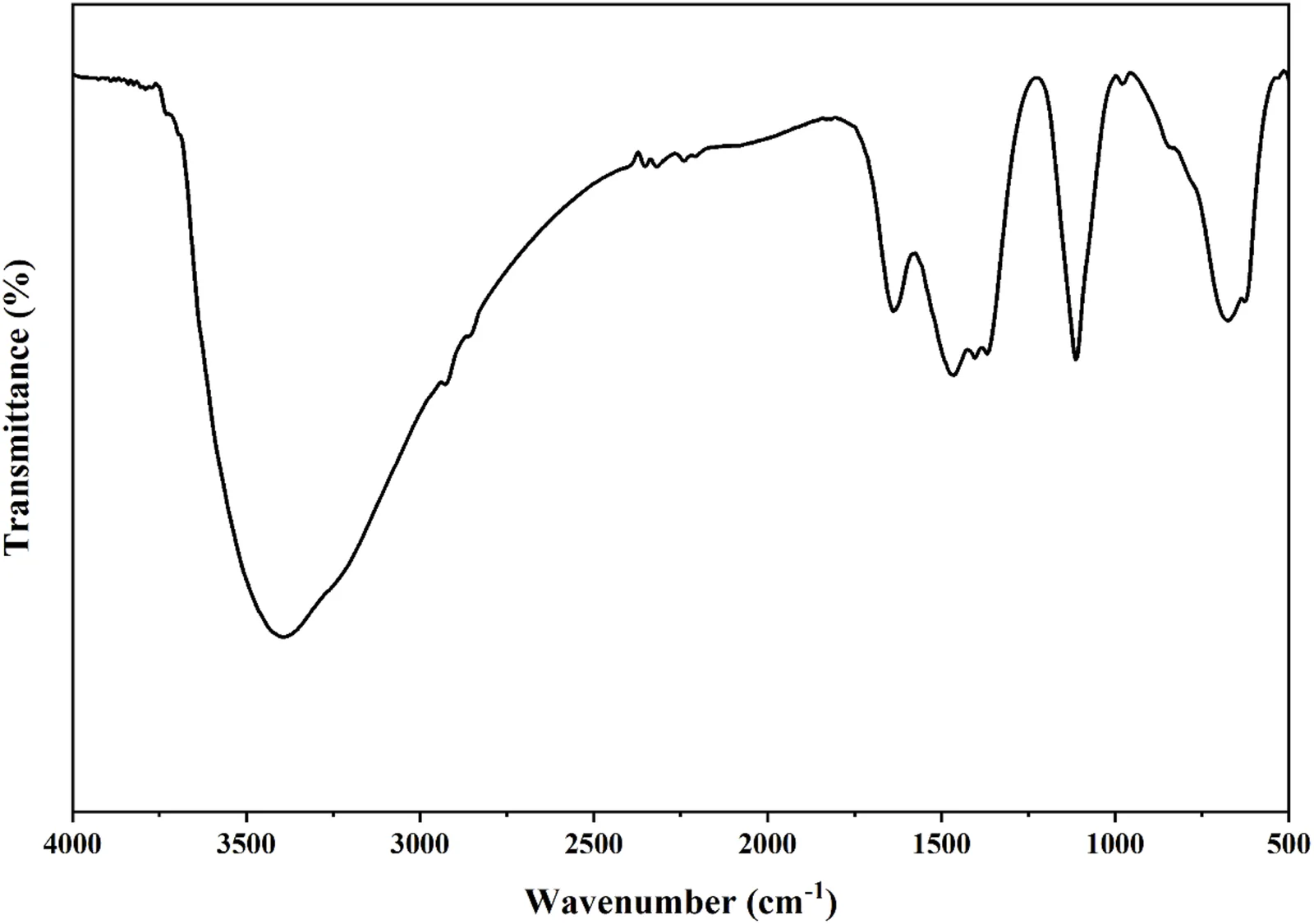
The FTIR spectra of LDH–NILq.

#### 3.4.1 Spectral features of LDH–NILq composite

The FTIR spectrum of the LDH–NILq composite (Fig. 4) exhibits a combination of characteristic absorption bands from both the LDH framework and the NILq component, confirming successful incorporation of NILq into the LDH structure. The spectrum displays a broad and intense absorption band centered at approximately 3392 cm^−1^, which is attributed to the overlapping O–H stretching vibrations of the LDH hydroxyl layers and interlayer water molecules, along with the O–H and N–H stretching vibrations of the chitosan matrix and the phenolic OH group of the quinoline-based ionic liquid. A weak absorption band is observed at approximately 2318 cm^−1^ in the LDH–NILq composite spectrum. This band is not typically observed in the free NILq spectra and may be attributed to several possible origins. In quaternary ammonium salts, combination bands or overtone vibrations of the C–N^+^ stretching modes can appear in this region. Additionally, the band may arise from the stretching vibration of the aromatic C–H bonds of the quinoline ring in the quinolinium cation, which can be enhanced upon charge delocalization in the ionic liquid structure. The presence of this band provides further evidence for the successful incorporation of the quinolinium ionic liquid into the LDH framework and may suggest some degree of electronic interaction between the quinolinium cation and the LDH surface. A weak band observed at approximately 1637 cm^−1^ corresponds to the O–H bending vibration of interlayer water molecules within the LDH structure. The presence of NILq is confirmed by the appearance of characteristic bands at approximately 1464 cm^−1^ and 1402 cm^−1^, assigned to the aromatic C=C stretching vibration of the quinoline ring and the C–N stretching vibration of the quinolinium nitrogen, respectively. Additionally, a band at approximately 1367 cm^−1^ corresponds to the C–O stretching vibration of the phenolic OH group of NILq, while the band at approximately 1113 cm^−1^ is assigned to the C–O–C stretching vibration of the chitosan backbone. A series of absorption bands in the low-frequency region between 400 and 700 cm^−1^ are assigned to M–O and M–OH lattice vibrations (Ni–O, Al–O, Ni–OH, and Al–OH stretching and bending modes), confirming the preservation of the LDH layered structure after NILq incorporation.

#### 3.4.2 Comparative analysis: NILq *vs.* LDH–NILq composite

Comparison of the FTIR spectrum of the LDH–NILq composite (Fig. 4) with that of free NILq (Fig. 3) reveals several important spectral modifications that provide direct evidence for chemical interactions between NILq and the LDH framework.

The broad OH/NH stretching band of NILq, observed in the region 3400–2980 cm^−1^ (Fig. 3), exhibits a noticeable shift to lower wavenumbers (centered at approximately 3392 cm^−1^) and significant broadening in the LDH–NILq composite (Fig. 4). This spectral change is attributed to the formation of hydrogen bonds between the hydroxyl groups of the LDH layers and the phenolic OH groups of NILq, the LDH surface hydroxyls and the NH/OH groups of the chitosan matrix, and the LDH hydroxyls and the quinoline nitrogen. This shift to lower wavenumbers with broadening is a characteristic signature of hydrogen bond formation and is consistent with previous studies reporting hydrogen bonding between ionic liquids and LDH surfaces.

Furthermore, the aromatic C=C stretching band of the quinoline ring, observed at approximately 1464 cm^−1^ in the LDH–NILq composite, shows a slight shift and intensity modification compared to the free NILq spectrum. Similarly, the C–N stretching band at approximately 1402 cm^−1^ exhibits detectable changes in position and relative intensity. These shifts suggest electrostatic interactions between the positively charged quinolinium cation and the negatively charged regions of the LDH framework, as well as between the π-electron system of the quinoline ring and the LDH surface. These electrostatic interactions are expected due to the highly positive surface charge of NILq (+27.8 mV, Section 3.2) and the charged nature of the LDH layers.

Additionally, the C–O stretching band of the phenolic OH group at approximately 1367 cm^−1^ and the C–O–C stretching band of the chitosan backbone at approximately 1113 cm^−1^ exhibit subtle shifts in the LDH–NILq composite compared to the free NILq spectrum, indicating that the chitosan matrix of the NILq nano-formulation also participates in the interaction with the LDH surface, likely through hydrogen bonding between the chitosan OH/NH groups and the LDH hydroxyls.

Importantly, the characteristic LDH bands—including the O–H stretching at ∼3392 cm^−1^, the interlayer water bending at ∼1637 cm^−1^, and the M–O/M–OH lattice vibrations at 400–700 cm^−1^, are well preserved in the LDH–NILq composite spectrum. This confirms that the LDH layered structure remains intact after NILq incorporation, despite the chemical interactions occurring at the surface and interlayer regions.

The combined spectral evidence provides strong support for chemical interactions between NILq and the LDH framework, extending beyond mere physical adsorption. The shift and broadening of the OH/NH stretching band confirm the formation of hydrogen bonds between LDH hydroxyls and the functional groups of NILq (phenolic OH, quinoline nitrogen, and chitosan OH/NH). Importantly, the retention of the characteristic LDH bands confirms that the LDH structure is preserved, indicating that these chemical interactions occur at the surface and interlayer regions without compromising the structural integrity of the LDH framework.

### 3.5. Characterization of LDH–SA coatings

#### 3.5.1 Wettability of the prepared superhydrophobic coatings

The electrodeposition of the LDH in the absence and presence of nano-formulated ionic liquid on the steel substrate was applied under potentiostatic conditions at an applied voltage of 7.0 V for 6.0 min (optimum conditions). This process generated the necessary surface roughness. To achieve low surface energy for superhydrophobicity of the coats the samples were dipped in a 0.01 M ethanolic stearic acid solution for 15 min. As shown in Fig. 5, the water contact angle of LDH–SA was 157.3° and sliding angle was 1.0° that confirming its superhydrophobic nature. But in the presence of 10 ppm of NILq with LDH the contact angle increases to 159.4° and decreases in sliding angle to 1.0° which means enhanced performance of superhydrophobicity of the coat. By increasing the concentration of the NILq to 20 ppm with LDH the contact angle increases to 162.4° and decreases in sliding angle to 1.0°. Finally, at the optimum concentration of 30 ppm NILq with LDH the contact angle shows a significant increase to 166.5° and a significant decrease in sliding angle to 0.5°. These results demonstrate that the addition of NILq substantially improves the superhydrophobic properties of the LDH–SA coating.

**Fig. 5:**
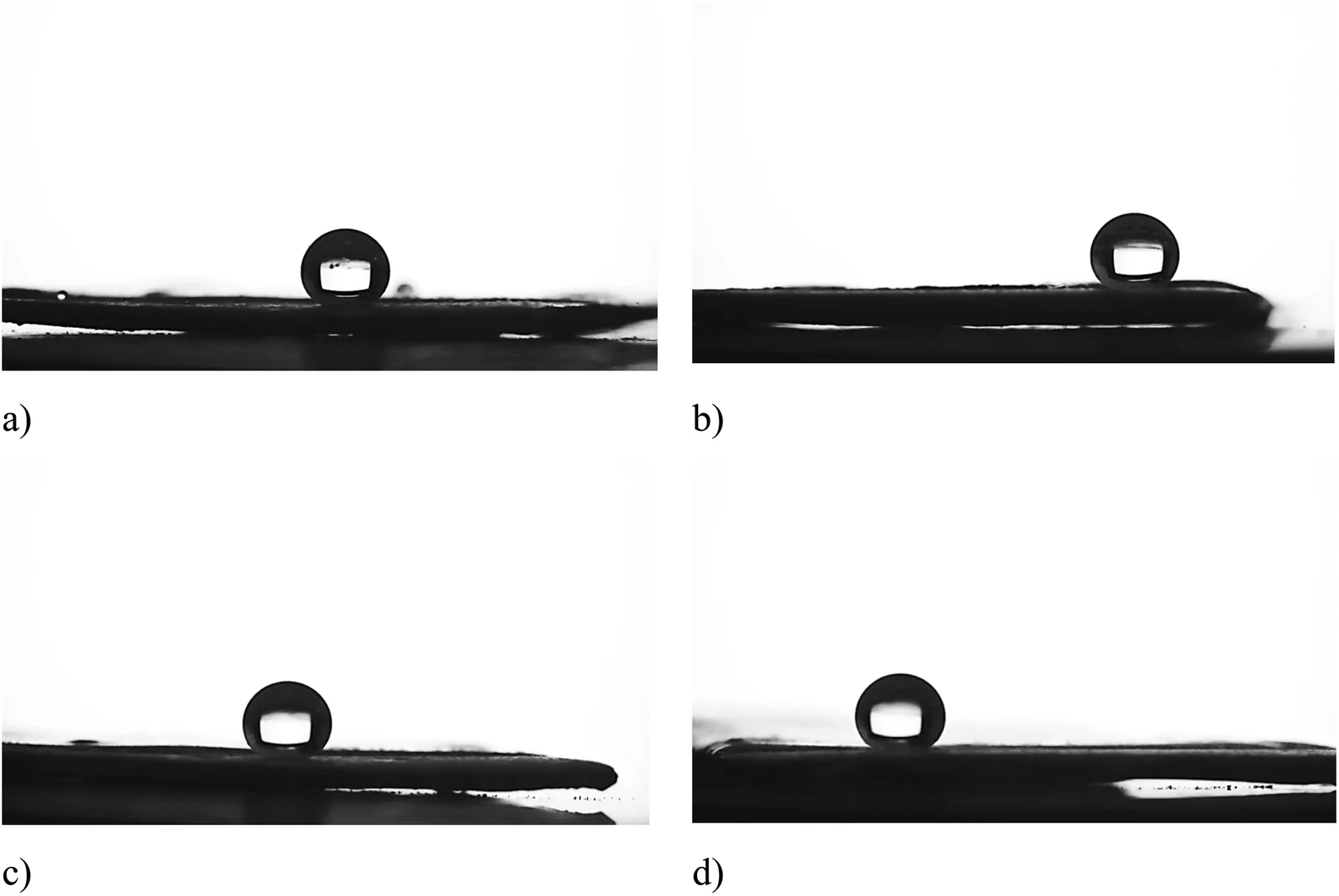
Water contact angles for (a) LDH–SA, (b) 10 ppm LDH–NILq–SA, (c) 20 ppm LDH–NILq–SA, and (d) 30 ppm LDH–NILq–SA.

#### 3.5.2 AFM and surface topography

The rough surface of the superhydrophobic coatings was characterized by atomic force microscopy (AFM), as shown in Fig. 6. The root means square roughness (*R*_a_) values were determined to evaluate the topographical evolution of the coatings upon incorporation of the NILq.

**Fig. 6:**
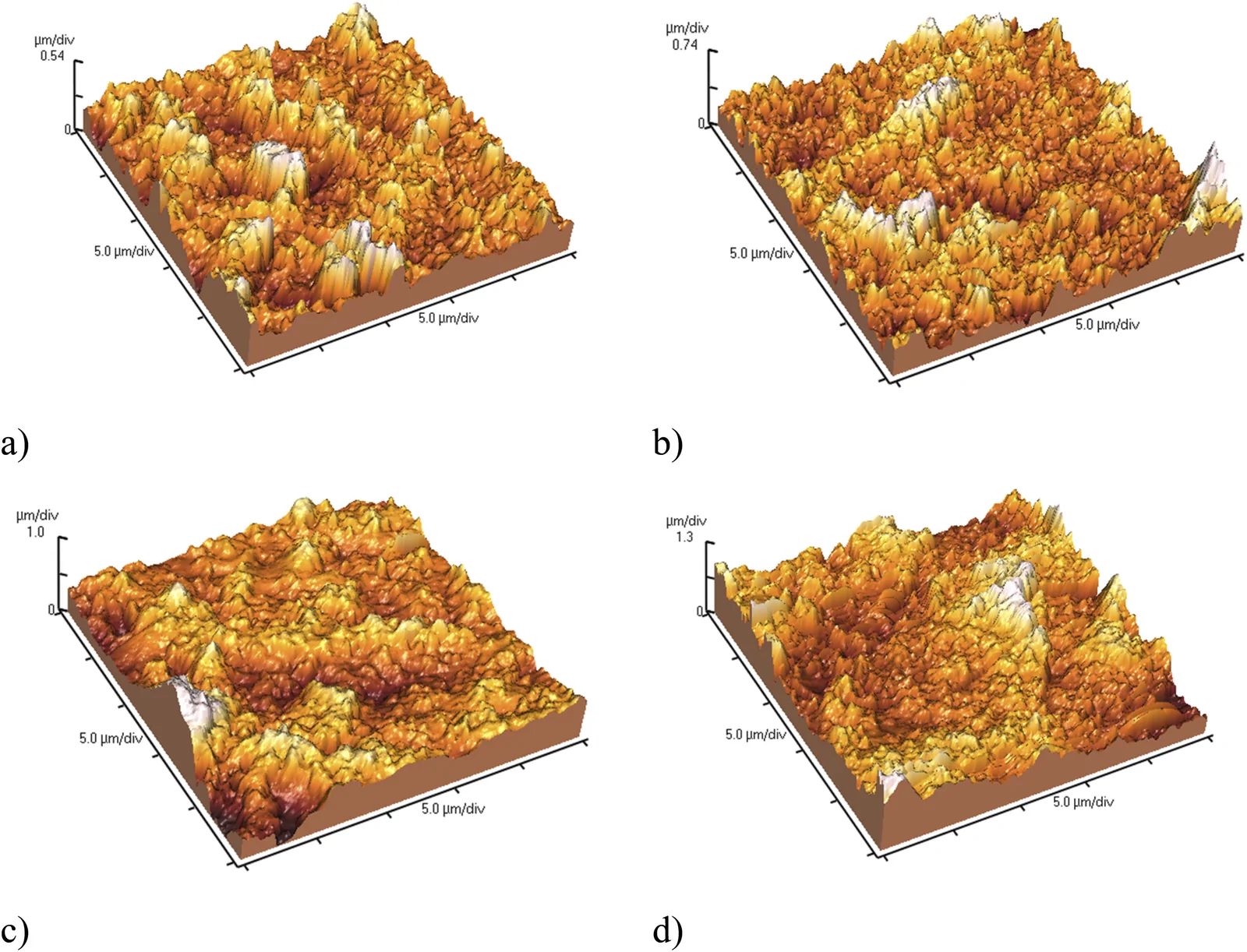
AFM nano-graphs of (a) LDH–SA, (b) 10 ppm LDH–NILq–SA, (c) 20 ppm LDH–NILq–SA, and (d) 30 ppm LDH–NILq–SA.

The LDH–SA coating (Fig. 6a) exhibited the lowest roughness value (*R*_a_ = 152.3 nm). In contrast, the sample prepared with 30 ppm NILq (30 ppm LDH–NILq–SA, Fig. 6d) displayed the highest roughness (*R*_a_ = 369.9 nm), indicating a marked enhancement in the active surface area and the development of a well-defined nanostructured surface architecture. The 20 ppm LDH–NILq–SA sample (Fig. 6c) showed an intermediate roughness (*R*_a_ = 241.2 nm), substantially higher than that of the pristine LDH–SA but lower than that of the 30 ppm sample. This result confirms the presence of a nanostructured layer; however, the topography appears not fully developed at this concentration, leading to a moderate enhancement in effective surface area. Meanwhile, the 10 ppm LDH–NILq–SA sample (Fig. 6b) exhibited a relatively low *R*_a_ value (172.5 nm), only marginally higher than that of LDH–SA, indicating limited nanoscale feature development and consequently reduced superhydrophobic potential. The observed trend clearly demonstrates that increasing the NILq concentration promotes the development of hierarchical surface roughness, with the 30 ppm sample achieving the most pronounced nanoscale features.

The correlation between surface roughness and superhydrophobic performance is well-established in the literature. According to the Cassie–Baxter model, surfaces with hierarchical micro–nano structures minimizing the solid–liquid contact area by trapping air pockets beneath water droplets which leading to low sliding angles and high water contact angles.^42^ The AFM results obtained in this study are consistent with this model. The progressive increase in surface roughness from 152.3 nm (LDH–SA) to 369.9 nm (30 ppm LDH–NILq–SA) directly corresponds to the enhanced wettability performance reported in Section 3.5.1, where the contact angle increased from 157.3° to 166.5° and the sliding angle decreased from 1.75° to 0.5°.

The relatively modest roughness of the 10 ppm LDH–NILq–SA sample (172.5 nm) explains its intermediate superhydrophobic performance, as the limited nanoscale features provide fewer air-trapping sites, allowing greater solid–liquid contact. Similarly, the incomplete development of nanostructures in the 20 ppm sample (241.2 nm) results in improved but not optimal performance. Only at the optimum concentration of 30 ppm does the surface achieve sufficient hierarchical complexity to maximize air entrapment, leading to the observed superhydrophobic enhancement.

These findings underscore the critical role of the nano-formulated ionic liquid in modulating the electrodeposition process. The presence of NILq influences nucleation and growth kinetics during LDH formation, promoting the development of finer and more complex surface features. This morphological evolution is essential for achieving the dual requirements of superhydrophobicity: sufficient surface roughness and the subsequent low-energy modification by stearic acid.

#### 3.5.3 Scanning electron microscopy (SEM)

Superhydrophobic coatings surface morphology was studied by scanning electron microscopy (SEM), as shown in Fig. 7. The LDH–SA coating prepared without NILq exhibits a well-defined layered structure characteristic of layered double hydroxides, with coarse nano-walls. This pronounced overgrowth, likely driven by Ostwald ripening during electrodeposition, results in relatively large structural features that limit the effective surface area and overall roughness.

**Fig. 7:**
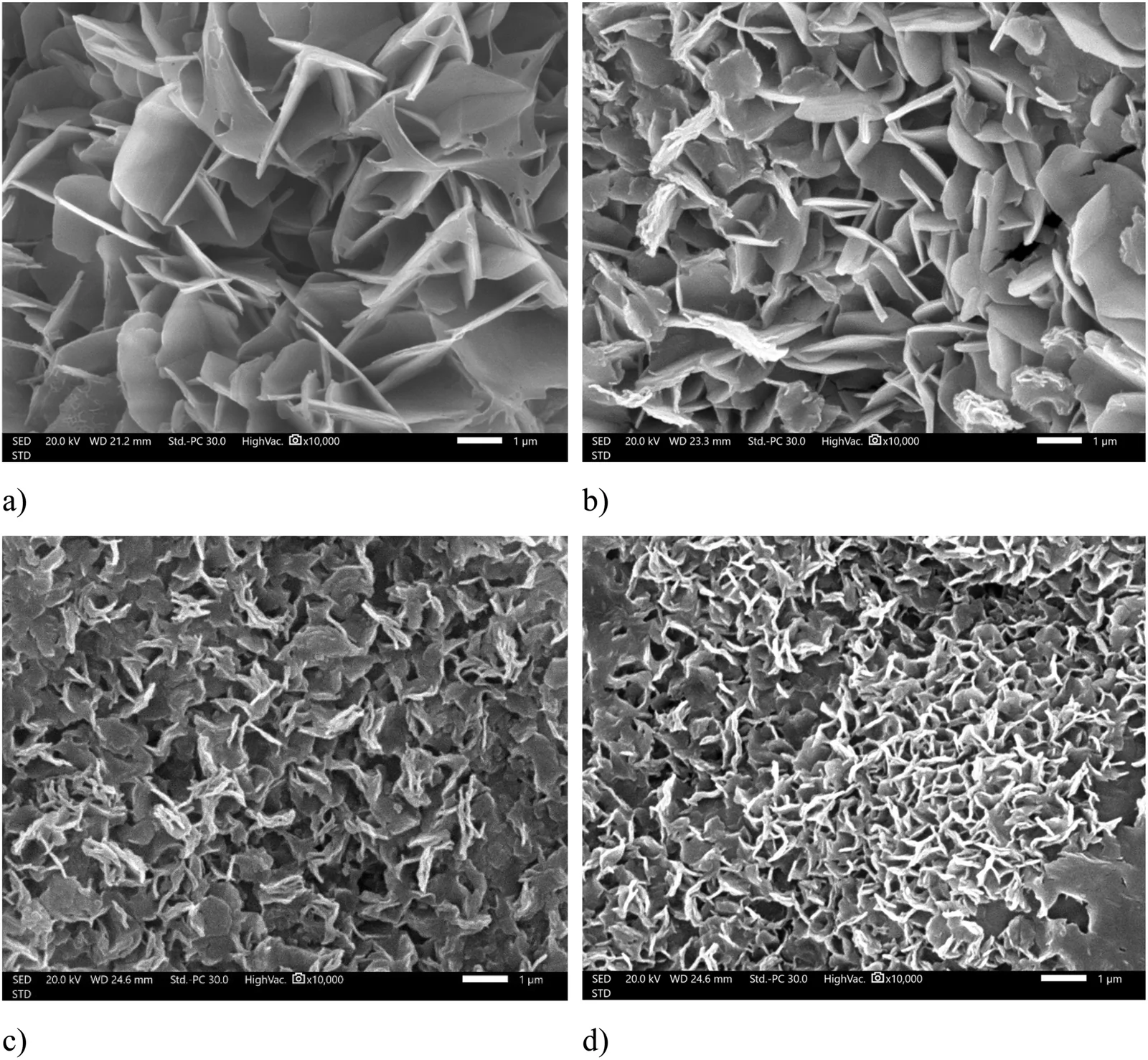
SEM of (a) LDH–SA, (b) 10 ppm LDH–NILq–SA, (c) 20 ppm LDH–NILq–SA, and (d) 30 ppm LDH–NILq–SA.

Upon incorporation of the nano-formulated ionic liquid, a systematic refinement of the LDH layered structure is observed. At a low NILq concentration (10 ppm LDH–NILq–SA), a modest decrease in the layer thickness occurs, producing sparsely distributed and poorly interconnected nano-walls with limited surface coverage. Although the layered structure begins to refine, the reduction in feature size is insufficient to generate the hierarchical roughness required for optimal superhydrophobicity.

Increasing the NILq concentration to 20 ppm (20 ppm LDH–NILq–SA) leads to a more pronounced reduction in the LDH layer dimensions, resulting in a well-developed network of fine nano-walls. This significant decrease in feature size enhances the surface area and roughness compared to the 10 ppm sample, yet the structure still lacks the optimal sharpness and density required for maximum air entrapment.

At the optimal NILq concentration of 30 ppm (30 ppm LDH–NILq–SA), a further refinement of the layered structure is achieved, producing a distinct nano-needle morphology. This represents the maximum reduction in LDH feature size, yielding the highest surface area and greatest surface roughness among all prepared coatings. The sharp, high-aspect-ratio architecture is particularly effective in generating hierarchical roughness and maximizing nanoscale air-trapping pockets.

This systematic progression-from coarse layered structures (0 ppm) to moderately refined layers (10 ppm), to significantly refined layers (20 ppm), and finally to optimally refined (30 ppm)-demonstrates that the nano-formulated ionic liquid acts as a structure-directing agent that progressively inhibits excessive crystal growth during electrodeposition. The result is a controlled reduction in LDH layer dimensions with increasing NILq concentration, leading to enhanced surface area, increased roughness, and consequently superior superhydrophobic performance at the optimal concentration of 30 ppm.

The morphological transition observed by SEM provides a structural basis for the wettability trends reported in Section 3.5.1. The Cassie–Baxter model effectively describes the relationship between surface architecture and superhydrophobicity, predicting that droplets rest on a composite solid–air interface, with the solid fraction controlling the contact angle and sliding behavior.

### 3.6. Chemical stability performance

The optimized LDH–NILq–SA (30 ppm) and LDH–SA coatings were evaluated across a pH range of 1–13 to provide the chemical stability, as shown in Fig. 8 and 9.

**Fig. 8:**
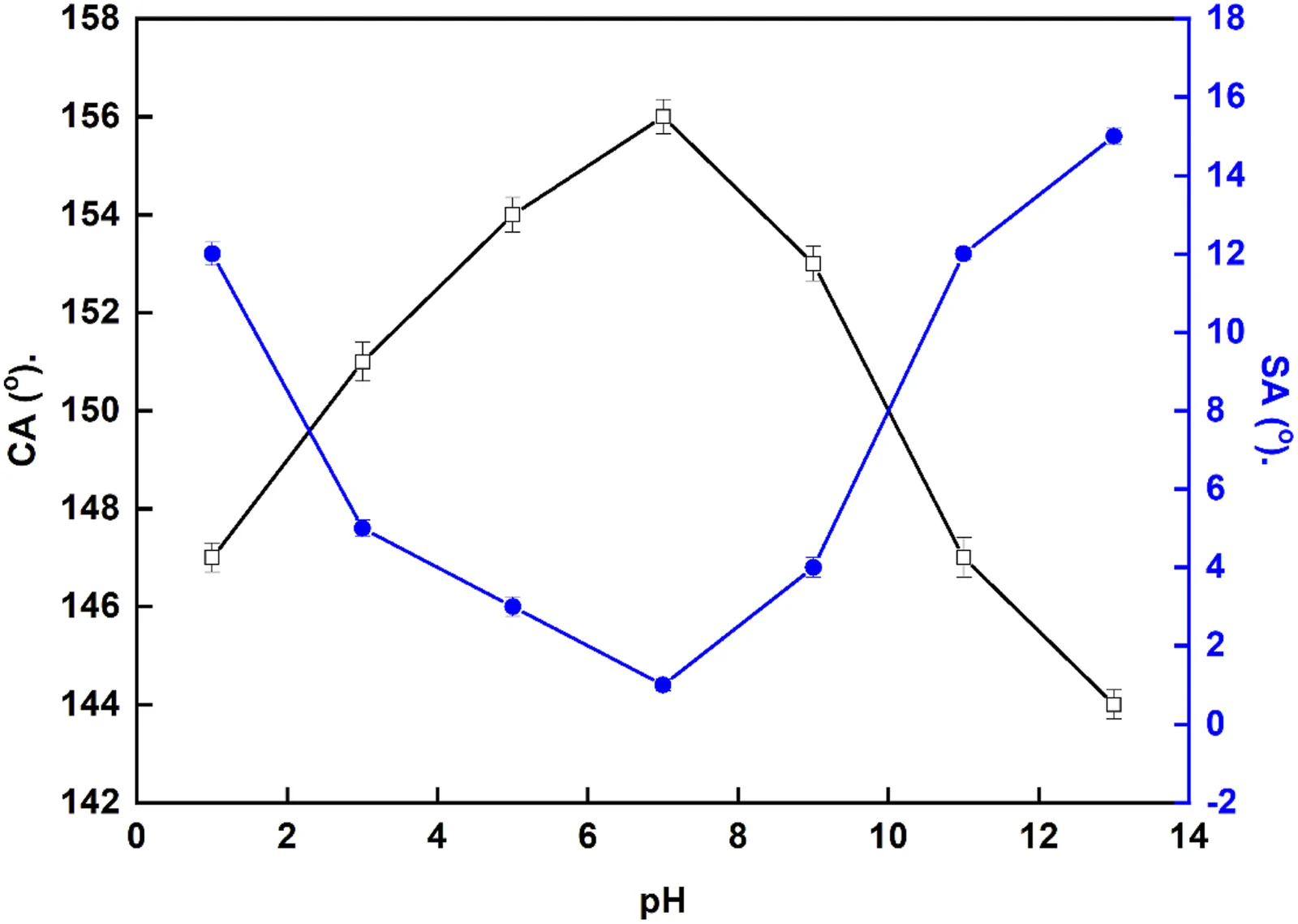
Variation of WCA and WSA on LDH–SA coatings following immersion in different pH environments.

**Fig. 9:**
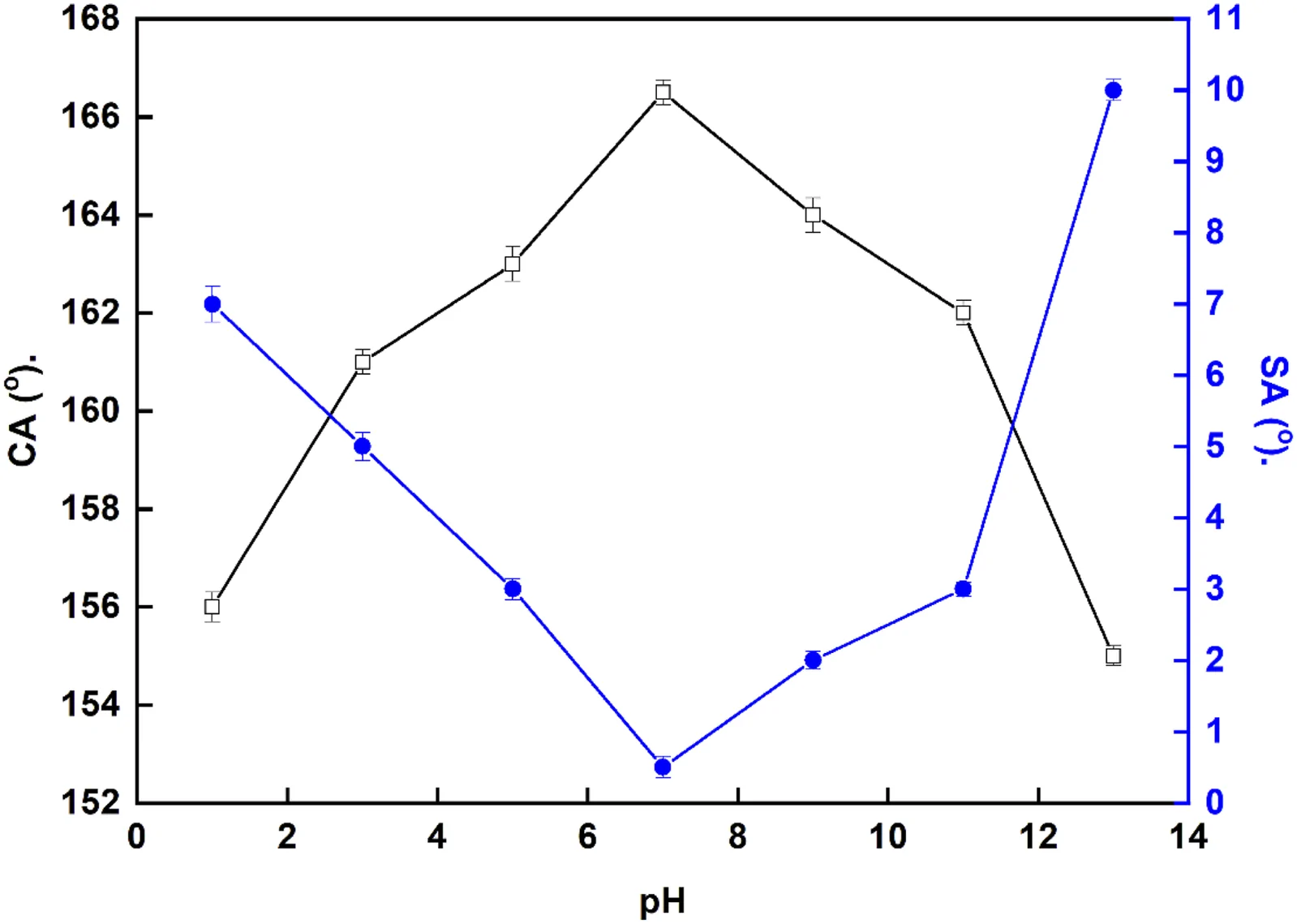
Variation of WCA and WSA on 30 ppm LDH–NILq–SA coatings following immersion in different pH environments.

As shown in Fig. 8 and 9, the LDH–SA coating exhibited noticeably higher SA and lower WCA compared to the LDH–NILq–SA coating across all pH values, indicating inferior chemical stability. The LDH–SA coating retained its SHP characteristics only within the pH of 3 to 9, Fig. 8. This degradation in performance suggests that the LDH–SA coating possesses limited stability, particularly in strong alkaline and acidic environments. The loss of superhydrophobicity can be attributed to significant structural degradation of the LDH layer under acidic and basic conditions, which compromises the surface roughness and disrupts the air-trapping mechanism essential for maintaining low adhesion and high-water repellency.

In the presence of the nano-formulated ionic liquid, the LDH–NILq–SA coating demonstrated markedly enhanced chemical stability. As shown in Fig. 9, the coating maintained its SHP characteristics over an extended pH from 1 to 13. These results demonstrate excellent LDH–NILq–SA coating chemical stability in acidic and basic circumstances.

The superior LDH–NILq–SA coating chemical stability compared to the LDH–SA coating due to numerous interrelated parameters. First, the addition of the nano-formulated ionic liquid promotes the formation of a more robust and well-defined nanostructure, as evidenced by the SEM and AFM results discussed in previous sections. The dense nano-needle morphology of the optimized coating (30 ppm NILq) provides enhanced mechanical integrity and resistance to chemical attack compared to the coarser nano-wall structure of the LDH–SA coating. Second, the strong chemical interactions between the nano-formulated ionic liquid and molecules of stearic acid with the metal cations within the structure of LDH lead to the formation of a stable low-surface-energy monolayer that is resistant to aggressive chemical conditions. The ionic liquid acts as a stabilizing agent, reinforcing the LDH framework and protecting it from dissolution or structural degradation upon exposure to extreme pH environments. Third, the preserved structural integrity of the LDH–NILq–SA coating under both acidic and alkaline conditions ensures the maintenance of the air-trapping capability essential for sustaining superhydrophobic performance. In contrast, the LDH–SA coating undergoes significant structural degradation at high and low pH, leading to the collapse of the hierarchical roughness and the loss of the Cassie–Baxter wetting state.

These findings confirm that the incorporation of the nano-formulated ionic liquid not only enhances the initial superhydrophobic performance but also suggestively enhances the durability of the coating under aggressive chemical environments, which is critical for practical applications in industrial and marine settings.

### 3.7. Mechanical stability performance

The mechanical stability of the superhydrophobic coatings was assessed *via* an abrasion resistance test. The LDH–SA coating maintained its SHP characteristics till the length of abrasion equals 350 mm. As shown in Fig. 10, this abrasion length represents the maximum distance over which the LDH–SA coating preserved its superhydrophobicity (WCA > 150°, SA < 10°). Beyond this point, the coating gradually lost its surface integrity and superhydrophobic characteristics, indicating a moderate level of mechanical durability.

**Fig. 10:**
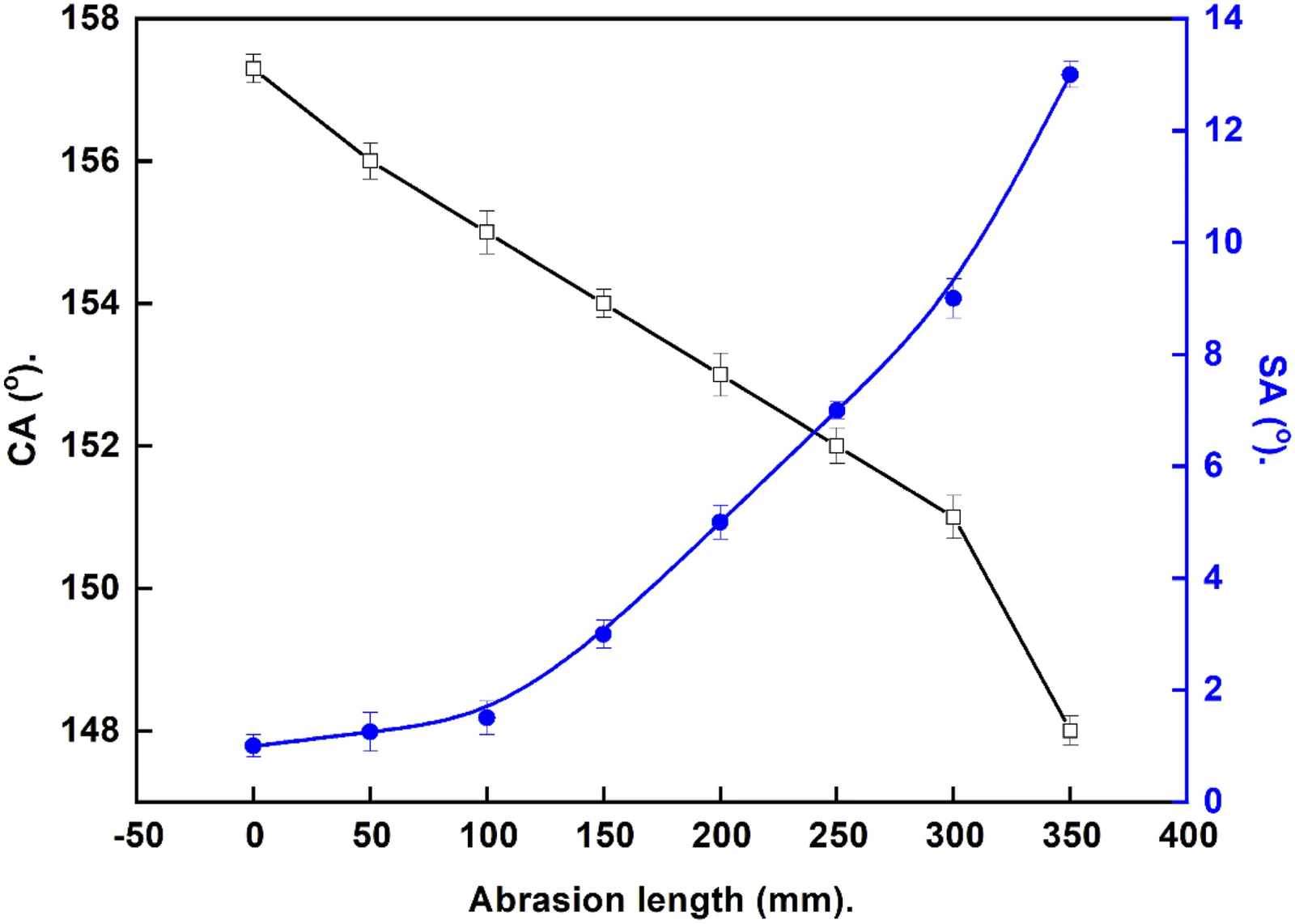
Variation of WCA and WSA as a function of abrasion length for the LDH–SA coating.

In the presence of the nano-formulated ionic liquid, the LDH–NILq–SA coating exhibited significantly enhanced mechanical durability. As shown in Fig. 11, the coating maintained its SHP characteristics till an abrasion distance of 1050 mm. This distance represents a threefold improvement over the NILq-free coating and indicates excellent mechanical resilience.

**Fig. 11:**
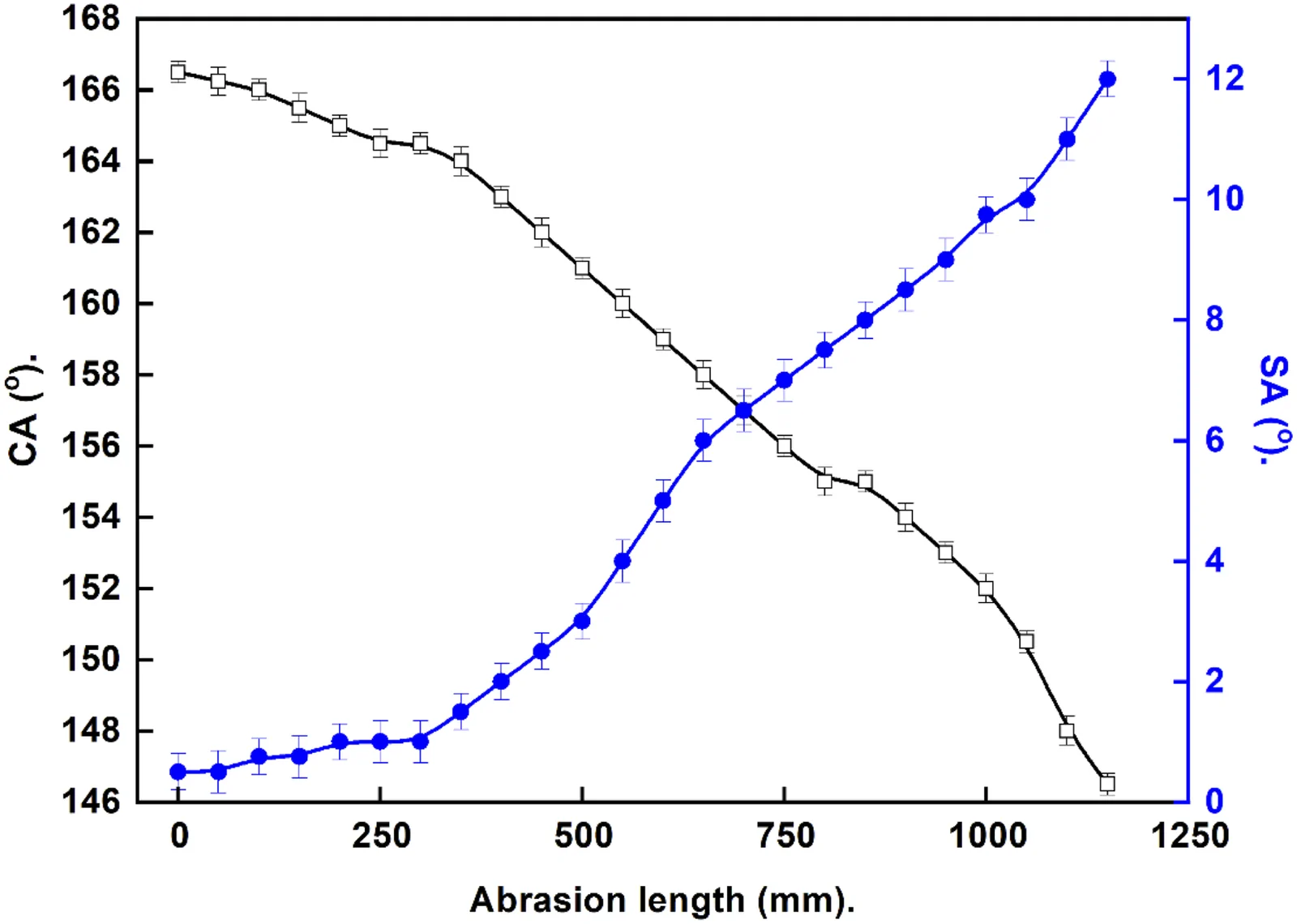
Variation of WCA and WSA as a function of abrasion length for the LDH–NILq–SA coating.

The threefold increase in abrasion resistance (from 350 mm to 1050 mm) demonstrates that the nano-formulated ionic liquid plays a critical role in enhancing the mechanical properties of the LDH coating. This improvement is likely due to the ionic liquid facilitating stronger interparticle bonding within the LDH matrix and promoting better interfacial adhesion with the substrate. The nano-needle architecture of the optimized coating may also contribute to mechanical robustness by distributing applied stress more effectively than the coarser nano-wall structure of the NILq-free coating.

The ability of the LDH–NILq–SA coating to withstand 1050 mm of abrasion while retaining superhydrophobicity represents a substantial advantage. This exceptional mechanical stability positions the developed coating as a practically viable material for real-world applications where surfaces are subjected to frequent contact, friction, or wear. Our coating withstands 1050 mm of linear abrasion under a 5 kPa load while maintaining superhydrophobicity, which is superior to many reported values for superhydrophobic coatings. For instance, a biomass-derived carbon quantum dot superhydrophobic coating retained its properties after 900 mm of abrasion.^43^ A superhydrophobic surface on magnesium alloy maintained superhydrophobicity after 900 mm of abrasion.^44^ The remarkable mechanical stability of the LDH–NILq–SA coating can be attributed to several factors. First, the strong adhesive interactions between the electrodeposited LDH–NILq layer and the steel substrate ensure robust anchoring of the coating to the underlying metal. Second, the incorporation of NILq contributes to the formation of a cohesive and well-integrated coating structure, as evidenced by the dense nano-needle morphology observed in SEM analysis (Section 3.5.3). Unlike fragile or powder-like layers that are easily delaminated, the NILq-containing coating exhibits structural integrity that resists mechanical abrasion.

The combination of excellent mechanical durability with the previously demonstrated chemical stability (Section 3.6) confirms that the LDH–NILq–SA coating is not merely a laboratory-scale demonstration but a technologically robust material. Its ability to maintain superhydrophobic performance under both mechanical stress and extreme pH conditions highlights its strong potential for practical applications, particularly in marine environments, industrial settings, and infrastructure exposed to harsh operational conditions.

### 3.8. Thermal stability performance

The thermal stability of the prepared coatings was evaluated by heating the samples across a temperature range of 150–250 °C in 50 °C increments, with each sample maintained at the target temperature for 1 h. The superhydrophobic performance was evaluated through measurements of WCA and SA after each thermal treatment, with superhydrophobicity defined as WCA > 150° and SA < 10°.

As shown in Fig. 12, the LDH–SA coating preserved its SHP characteristics till 150 °C, exhibiting a contact angle of 151.5° and a sliding angle of approximately 1°. However, upon increasing the temperature to 200 °C, localized regions of the LDH–SA coating transitioned to hydrophobic behavior, as indicated by an increase in sliding angle above 10° and a decrease in contact angle below 150°.

**Fig. 12:**
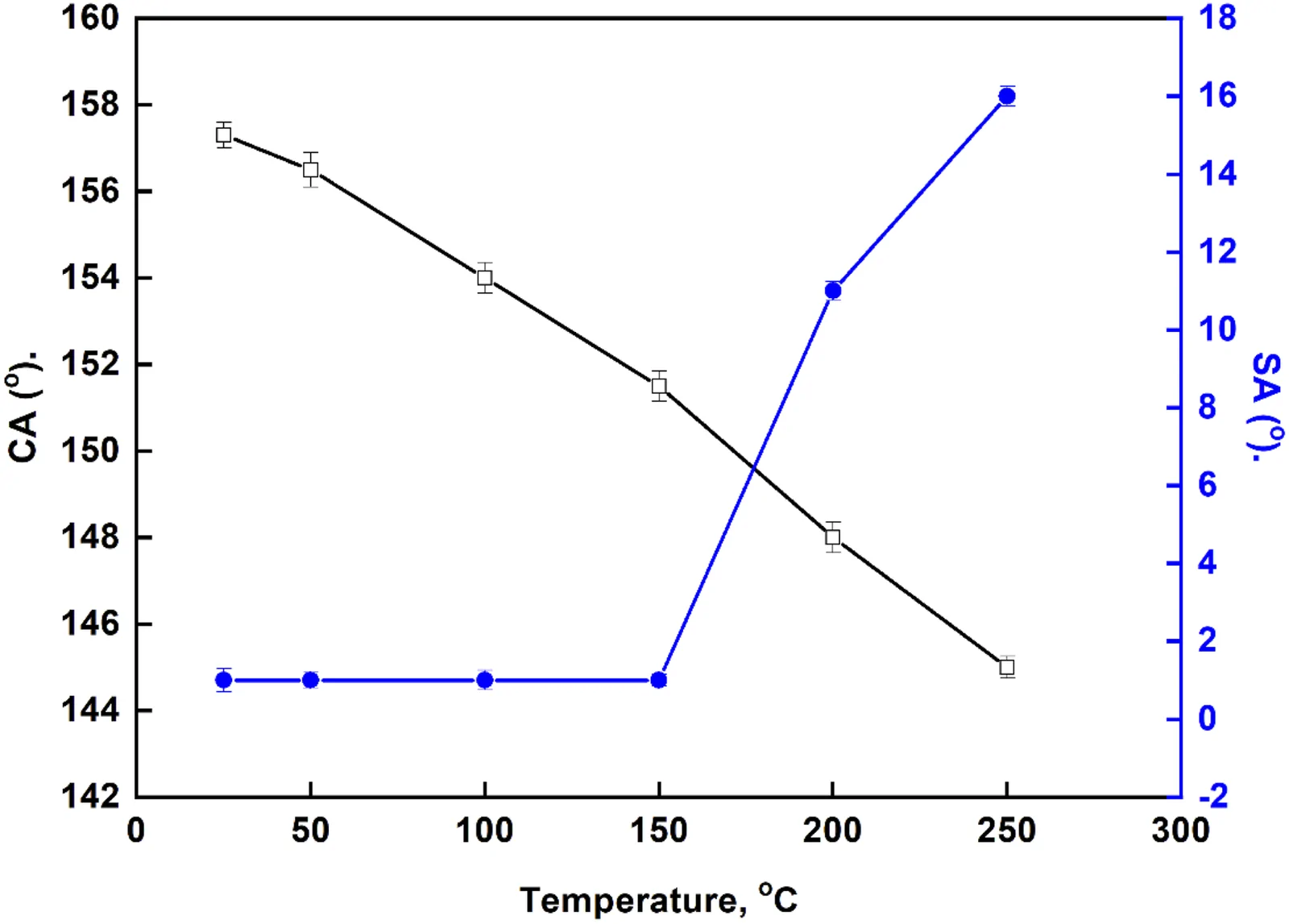
Variation of WCA and WSA as a function of temperature for the LDH–SA.

In contrast, the LDH–NILq–SA coating demonstrated enhanced thermal stability, as illustrated in Fig. 13. The coating maintained its superhydrophobic properties up to 200 °C, with a contact angle of 152° and a sliding angle of 0.5°. When the temperature reached 250 °C, localized areas of the LDH–NILq–SA coating began to lose superhydrophobicity, characterized by sliding angles exceeding 10° and contact angles falling below 150°.

**Fig. 13:**
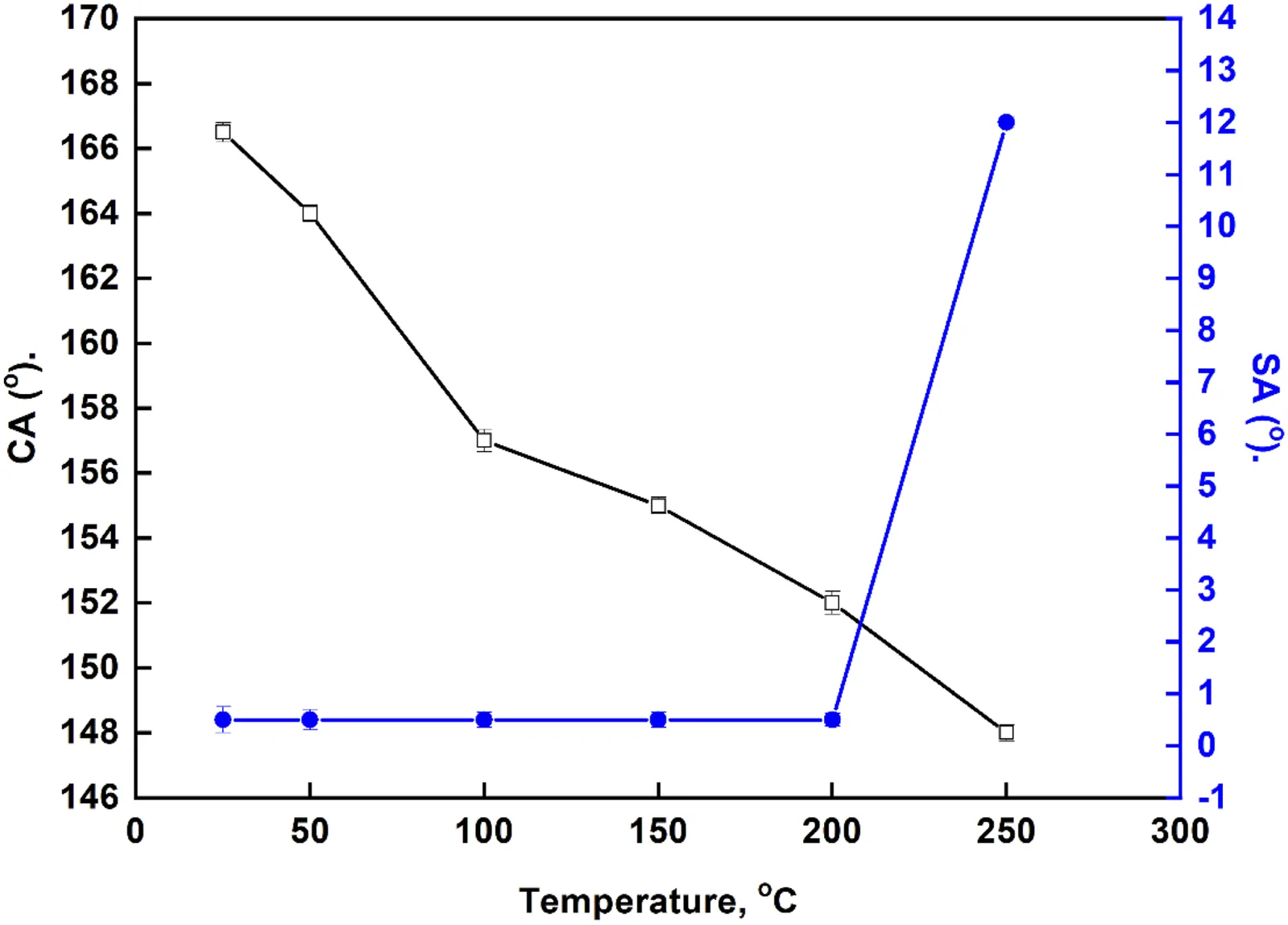
Variation of WCA and WSA as a function of temperature for the LDH–NILq–SA.

The observed degradation in superhydrophobic performance at elevated temperatures can be attributed to the partial decomposition or desorption of the stearic acid layer, which is responsible for the low surface energy of the coating. Prolonged exposure to high temperatures likely disrupts the bonding between the LDH surface and stearic acid molecules, compromising the integrity of the hydrophobic monolayer and consequently leading to the loss of the superhydrophobic state.

Notably, after re-immersion of both thermally degraded samples in a 1.0 M ethanolic stearic acid solution for 15 min, the superhydrophobic properties were effectively restored. This recovery demonstrates that the underlying LDH and LDH–NILq hierarchical structures remained intact after thermal exposure, and that the loss of superhydrophobicity was primarily due to stearic acid degradation rather than collapse of the surface architecture. The successful re-adsorption of stearic acid molecules onto the coating surface confirms the regenerability of these superhydrophobic coatings, which is advantageous for practical applications requiring long-term durability and maintenance.

The enhanced thermal stability of the LDH–NILq–SA coating compared to the NILq-free LDH–SA coating attributed to the role of the nano-formulated ionic liquid in strengthening the interaction between the stearic acid modifier and the LDH substrate. The highly positive surface charge of NILq (+27.8 mV) may facilitate stronger electrostatic interactions with the carboxylate groups of stearic acid, creating a more thermally robust hydrophobic layer. Additionally, the small size architecture roughness provides greater surface area for stearic acid adsorption, potentially delaying thermal desorption.

The restoration capability demonstrated here is significant, as it indicates that the superhydrophobic coating can be regenerated *in situ* without requiring complete re-fabrication. This feature enhances the practical viability of the coating for industrial applications where periodic maintenance is feasible.

### 3.9. Corrosion rate measurements

#### 3.9.1 Electrochemical impedance spectroscopy technique

The electrochemical impedance spectroscopy (EIS) results provide a comprehensive assessment of the corrosion protection performance of the prepared superhydrophobic coatings, showing strong agreement with the previously discussed wettability and structural analyses. Fig. 14 presents the Bode and Nyquist diagrams for uncoated and the superhydrophobic coated steel in a solution of 0.5 M NaCl.

**Fig. 14:**
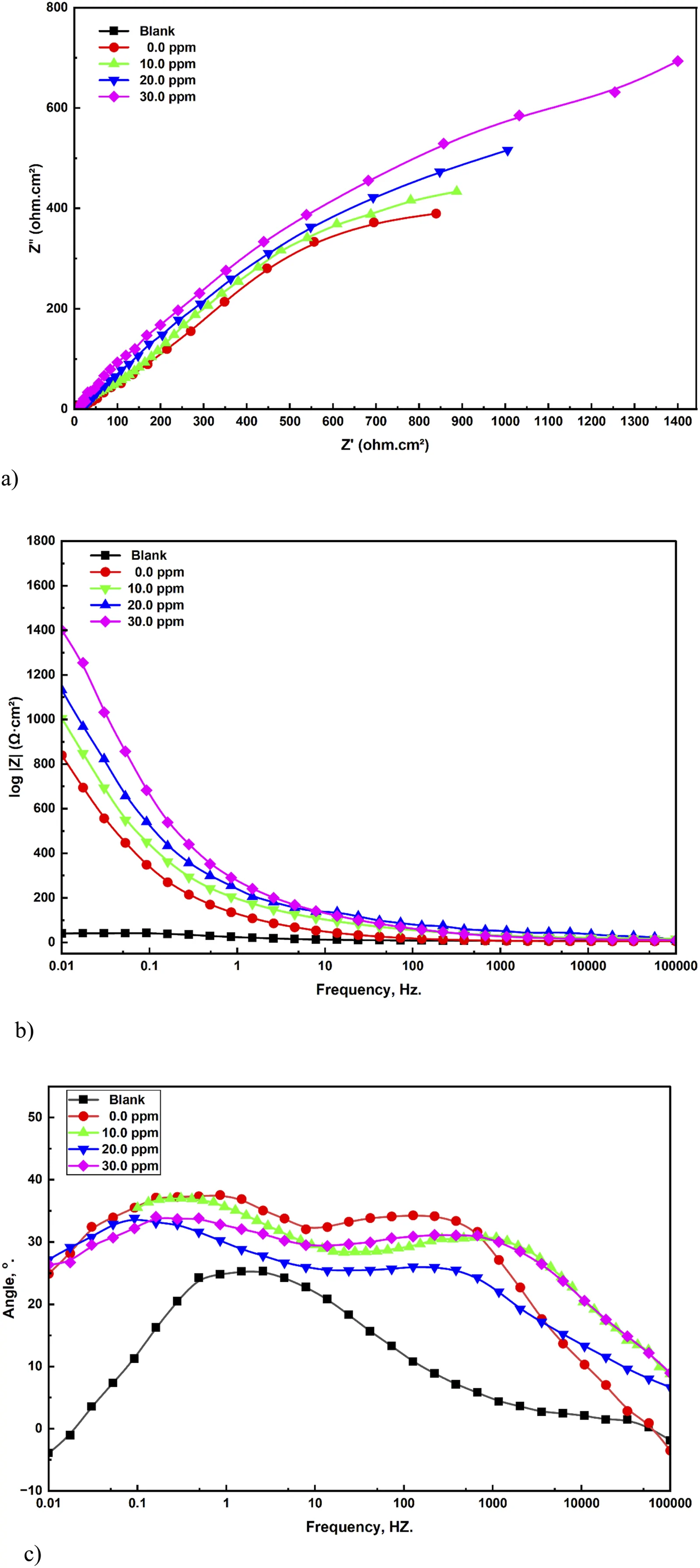
Nyquist (a), Bode magnitude (b), and phase angle (c) plots for bare steel and SHP coated steel after immersion in a solution of 0.5 M NaCl.

The Nyquist diagrams (Fig. 14a) display depressed capacitive semicircles, which are typical of charge-transfer processes occurring at the interface of electrode/electrolyte.^45^ Notably, the superhydrophobic-coated samples display significantly larger semicircle diameters compared to bare steel. Since the semicircle diameter directly equals to the charge transfer resistance (*R*_ct_), a higher *R*_ct_ indicates increased resistance to electrochemical corrosion reactions and slower corrosion kinetics.

Bare steel shows the lowest *R*_ct_ value, reflecting its poor corrosion resistance in chloride-containing media. The LDH–SA coating shows a higher *R*_ct_ value compared with bare steel, confirming that the LDH–SA layer functions as a physical barrier that partially hinders the diffusion of water and corrosive chloride ions toward the surface of steel. Incorporation of the nano-formulated ionic liquid further enhances corrosion resistance, as the 10 ppm LDH–NILq–SA coating displays a higher *R*_ct_ than the LDH–SA coating, which can be attributed to its improved superhydrophobicity. A continued increase in NILq concentration results in further enhancement, with the 20 ppm LDH–NILq–SA coating outperforming the 10 ppm sample. The 30 ppm LDH–NILq–SA coating (optimal concentration) exhibits the highest *R*_ct_ value, providing definitive evidence of its superior corrosion protection performance.

This outstanding behavior is attributed to the formation of a dense, uniform, and finely structured LDH–NILq layer with optimal surface roughness, combined with excellent superhydrophobicity. The high WCA (166.5°) promotes stable air entrapment within the surface nanostructure, forming an additional insulating layer that effectively suppresses electrolyte penetration and subsequent corrosion processes.

The Bode modulus plots (Fig. 14b) reveal that the impedance modulus at low frequencies (|*Z*|_0.01_ Hz), an indicator of the overall barrier efficiency of the coating, follows the same trend observed for *R*_ct_:bare steel < LDH–SA < 10 ppm LDH–NILq–SA < 20 ppm LDH–NILq–SA< 30 ppm LDH–NILq–SA.

Higher low-frequency impedance values correspond to superior corrosion protection, thereby quantitatively corroborating the Nyquist plot analysis.

The Bode phase angle plots (Fig. 14c) reveal the presence of two distinct time constants. The time constant observed at intermediate frequencies associated with the response of the superhydrophobic coating layer, while the time constant at low frequencies is associated with the electrochemical double layer formed at the interface of metal-solution.^46,47^

The phase-angle response is sensitive to surface morphology, porosity, and the degree of overlap between the two time constants. The 20 ppm NILq sample, being in a transitional morphological state between the sparse nano-walls of the 10 ppm NILq sample and the dense nano-needles of the 30 ppm NILq sample, exhibits a slightly different phase-angle profile. Nevertheless, the quantitative metrics – *R*_ct_, log|*Z*| at low frequency, and protection efficiency – all show a consistent monotonic improvement with increasing NILq concentration, confirming the progressive enhancement of corrosion protection.

The coated samples exhibit a maximum phase angle of approximately 35°, which is typical of protective coatings with a porous structure. A phase angle below 90° reflects non-ideal capacitive manners, which can be attributed to surface coating porosity and heterogeneity.

The deviation from ideal capacitor behavior (90°) observed here suggests a distributed capacitive response caused by the inherent roughness and porosity of the LDH-based coating, which is consistent with the use of a constant phase element (CPE) in the equivalent circuit model.

To further quantify the corrosion protection performance, the EIS data were fitted *via* the equivalent electrical circuit displayed in Fig. 15, and the impedance parameters were extracted *via* Zsimpwin program. The equivalent circuit consists of the solution resistance (*R*_s_), coating resistance (*R*_coat_), double-layer constant phase element (CPE_dl_), charge transfer resistance (*R*_ct_), and coating constant phase element (CPE_coating_). The protection efficiency (% *P*) was estimated *via*eqn (4):^48^

4 % *P* = [(*R*_ct_ − *R*_ct0_)/*R*_ct_] × 100

where *R*_ct0_ and *R*_ct_ represent the charge transfer resistance of bare steel and SHP coated steel, respectively.

**Fig. 15:**
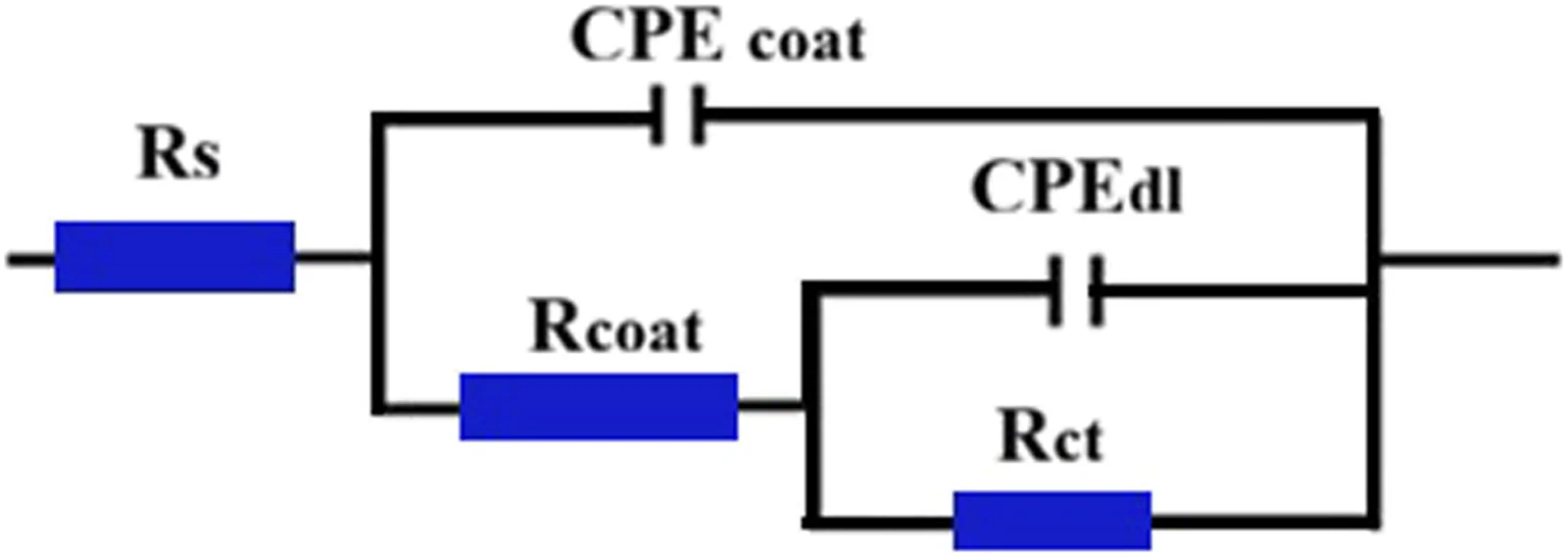
The equivalent circuit model.

The fitted impedance parameters are summarized in Table 1 the *R*_ct_ values and the calculated protection efficiencies are obey the following trend:bare steel < LDH–SA < 10 ppm LDH–NILq–SA < 20 ppm LDH–NILq–SA< 30 ppm LDH–NILq–SA,

**Table 1:** Impedance parameters for bare steel and the LDH superhydrophobic coating in 0.5 M NaCl solution

Substrate	*R*_s_ (Ohm cm^2^)	CPE_coat_ × 10^5^ (S^*n*^ Ω^−1^ cm^2^)	*n*_1_	*R*_coat_ (Ohm cm^2^)	CPE_dl_ × 10^5^ (S^*n*^ Ω^−1^ cm^2^)	*n*_2_	*R*_ct_ (Ohm cm^2^)	% *P*	Chi squared
Bare steel	1.2	463.4	0.64	2.17	2772.0	0.67	33.6	—	2.0 × 10^−4^
0.0 ppm NILq	4.8	2.6 × 10^−3^	0.44	18.3	2.5 × 10^−5^	0.62	2809	98.80	1.0 × 10^−3^
10.0 ppm NILq	11.8	1.9 × 10^−4^	0.56	145.4	9.2 × 10^−4^	0.52	5431	99.38	7.3 × 10^−5^
20.0 ppm NILq	12.1	2.0 × 10^−3^	0.58	181.3	3.8 × 10^−4^	0.50	6009	99.44	5.8 × 10^−4^
30.0 ppm NILq	17.6	1.8 × 10^−4^	0.52	311.0	5.9 × 10^−4^	0.48	6848	99.51	3.5 × 10^−4^

This progressive improvement in corrosion resistance with increasing NILq concentration confirms that the nano-formulated ionic liquid plays a crucial role in enhancing the barrier properties of the LDH coating.

The EIS measurements in 0.5 M NaCl solution demonstrated that the optimized LDH–NILq–SA coating achieved an exceptional corrosion protection efficiency of 99.5%. This value is notably superior to several recently reported superhydrophobic coatings evaluated under similar conditions. For instance, LDH-based superhydrophobic coating on steel fabricated *via in situ* electrodeposition achieved a protection efficiency of 98.5%,^49^ while a biomass-derived carbon quantum dot superhydrophobic coating on steel exhibited an efficiency of 93.9%.^43^ Furthermore, a plasma-sprayed CeO_2_ superhydrophobic coating on SS304 steel showed a protection efficiency of 97.1%.^50^ The superior performance of our LDH–NILq–SA coating can be attributed to the synergistic combination of the hierarchical micro-nano roughness imparted by the LDH structure, the low surface energy provided by stearic acid modification, and the enhanced structural integrity and air-trapping capability conferred by the incorporation of the nano-formulated quinoline-based ionic liquid (NILq). This combination effectively minimizes the solid–liquid contact area, thereby significantly impeding the diffusion of corrosive chloride ions and suppressing electrochemical corrosion reactions at the steel–electrolyte interface.

#### 3.9.2 Potentiodynamic polarization technique

The PDP curves provide quantitative insight into the corrosion kinetics and protective efficiency of the prepared superhydrophobic coatings. As shown in Fig. 16, all superhydrophobic-coated samples exhibit a significant improvement in resist corrosion compared to bare steel. This enhancement is indicated by a shift of the corrosion potential (*E*_corr_) toward more noble (positive) values, along with a distinct displacement of both the cathodic and anodic polarization branches toward lower current density regions. These shifts confirm that the coatings significantly suppress both anodic dissolution metal and cathodic reduction oxygen reactions, which are the primary driving forces of the corrosion process. Notably, the cathodic branches of the coated samples display a limiting current behavior, indicative of diffusion-controlled oxygen reduction through the superhydrophobic layer.

**Fig. 16:**
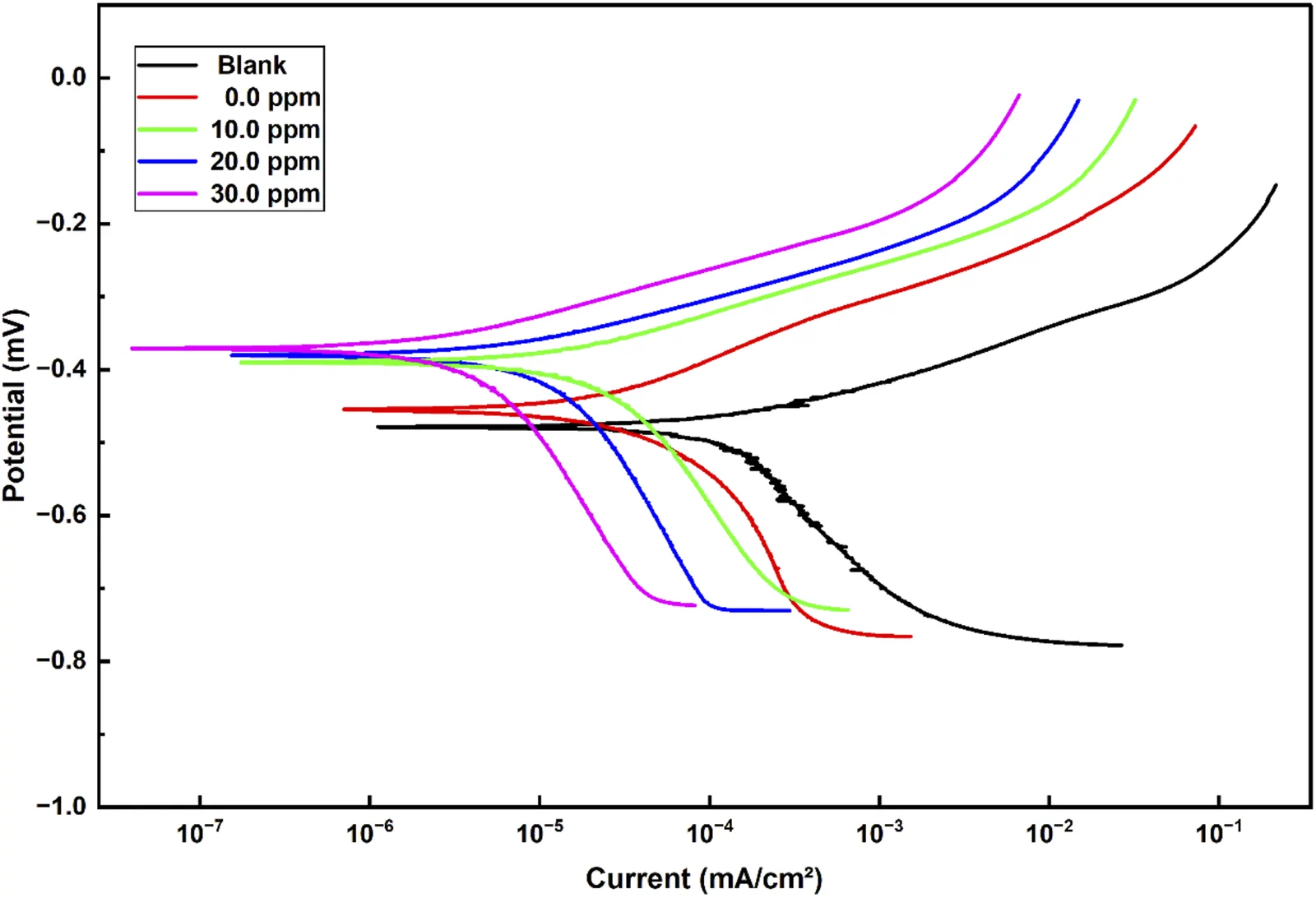
The PDP plots for bare and SHP coated steel in a solution of 0.5 M NaCl.

The extracted PDP parameters, including corrosion current density (*i*_corr._), anodic and cathodic Tafel slopes (*β*_a_ and *β*_c_), corrosion potential (*E*_corr._), and protection efficiency (% *P*), are summarized in Table 2. The protection efficiency was determined using eqn (5):^48^

5 % *P* = [(*i*^0^_corr._ − *i*_corr._)/*i*^0^_corr._] × 100

where *i*^0^_corr._ and *i*_corr._ represent the corrosion current densities of bare steel and the superhydrophobic-coated steel, respectively.

**Table 2:** The PDP parameters for bare steel and the LDH superhydrophobic coating in a 0.5 M NaCl solution

Coat	−*E*_corr._ mV	*β*_a_ mV per decade	−*β*_c_ mV per decade	*i*_corr._ µA cm^−2^	% *P*
Bare steel	589.6	84.8	232.8	388.80	—
0.0 ppm NILq	454.5	110.2	260.9	36.38	90.6
10.0 ppm NILq	380.0	76.9	343.6	21.47	94.48
20.0 ppm NILq	389.6	95.4	331.2	15.27	96.07
30.0 ppm NILq	371.0	86.7	359.3	12.14	96.88

As expected, all superhydrophobic-coated samples exhibit markedly lower *i*_corr._ values than bare steel, confirming their effective corrosion protection. The corrosion current densities follow the order:30 ppm LDH–NILq–SA < 20 ppm LDH–NILq–SA < 10 ppm LDH–NILq–SA < LDH–SA < bare steel

This trend indicates a progressive increase in corrosion resistance with increasing NILq concentration. Among all samples, the 30 ppm LDH–NILq–SA coating (optimal concentration) demonstrates the lowest *i*_corr._ and the most positive *E*_corr._, reflecting the greatest inhibition of both cathodic and anodic processes. The 20 ppm LDH–NILq–SA coating provides intermediate protection, followed by the 10 ppm LDH–NILq–SA coating, while the LDH–SA coating exhibits the lowest protection efficiency among the coated samples.

The substantial reduction in corrosion current density indicates that the superhydrophobic coat behaves as an efficient barrier against the ingress of chloride ions. Moreover, the exceptionally low *i*_corr._ values observed for the 30 ppm LDH–NILq–SA coating indicates that the entrapped air layer within the hierarchical surface architecture contributes extra electrical insulation, further impeding charge transfer reactions.

In addition, the Tafel analysis reveals a shift of the pitting potential (*E*_pit_) toward more positive values for the coated samples, indicating enhanced resistance to localized corrosion.^51^ This behavior is fully consistent with the corrosion protection trends obtained from the EIS measurements.

The polarization results corroborate the EIS findings and provide complementary vision into the corrosion inhibition mechanism. The simultaneous displacement of both cathodic and anodic parts signifies that the superhydrophobic coating functions as a mixed-type inhibitor, retarding both half-cell reactions without preferentially suppressing one over the other. The observation of limiting current behavior in the cathodic branches of coated samples is particularly significant. This diffusion-limited response suggests that the oxygen reduction reaction-the primary cathodic process in neutral aerated NaCl solution-is hindered by restricted oxygen transport through the superhydrophobic layer. The entrapped air layer within the hierarchical nanostructure effectively reduces the available pathways for oxygen diffusion to the steel surface, thereby slowing the overall corrosion kinetics.

The progressive improvement in corrosion resistance with increasing NILq concentration correlates directly with the enhanced superhydrophobicity and optimized surface morphology discussed in previous sections. The 30 ppm LDH–NILq–SA coating, with its dense nano-needle architecture and highest water contact angle (166.5°), maximizes air entrapment and minimizes solid–liquid contact, resulting in the most effective corrosion barrier. The positive shift in pitting potential for the coated samples indicates that the superhydrophobic layer not only provides general corrosion protection but also delays the onset of localized attack, which is particularly valuable for applications in chloride-rich environments where pitting corrosion is a primary failure mechanism.

Based on our experimental characterization (SEM, AFM, zeta potential, superhydrophobic coat performance, and wettability tests) and recent literature on IL-LDH systems, the role of NILq can be rationalized through three interconnected mechanisms:

(1) Structure-directing/crystal growth modulation:

Recent studies have demonstrated that ionic liquids can act as structure-directing agents by coordinating with metal cations (Ni^2+^, Al^3+^), thereby delaying crystallization kinetics and promoting the formation of finer, more refined nanostructures. For instance, Wu *et al.* reported that ionic liquids delay the crystallization of Ni^2+^ions, promoting core–shell structures, while ionothermal synthesis methods have shown that ILs effectively expand interlayer spacing and control crystal morphology. In our system, the progressive morphological transition from coarse nano-walls (0 ppm) to dense nano-needles (30 ppm) observed by SEM and AFM directly confirm this structure-directing effect.^52,53^

(2) Interfacial adsorption and electrostatic modulation:

Our zeta potential measurements revealed a highly positive surface charge of +27.8 mV for NILq. During cathodic electrodeposition, the positively charged NILq adsorbs onto the cathode surface. This interfacial adsorption layer modulates the local electric field and provides steric hindrance, which effectively inhibits excessive crystal overgrowth (Ostwald ripening) and promotes high-density nucleation. This is why we observed the highest roughness (*R*_a_ = 369.9 nm) at the optimal 30 ppm concentration.^54,55^

(3) Synergistic stabilization for multifunctional durability:

The enhanced chemical, mechanical, and thermal stability of the LDH–NILq–SA coating is attributed to strong electrostatic/hydrogen-bonding interactions between the positively charged NILq, the LDH framework, and the carboxylate groups of stearic acid. This multi-point interaction reinforces the coating's structural integrity and the stability of the hydrophobic monolayer, which is consistent with recent reports on ionic liquid-modified LDH nanocontainers for active corrosion protection.^56^

A critical consideration for the long-term performance of ionic liquid-modified coatings is the potential migration or leaching of the ionic liquid component during prolonged aqueous exposure. In the present system, the NILq is expected to exhibit strong resistance to leaching due to multiple anchoring mechanisms: (i) electrostatic interactions between the positively charged NILq (+27.8 mV) and the LDH framework; (ii) hydrogen bonding between the phenolic OH groups of NILq and the LDH hydroxyl layers; (iii) physical confinement within the LDH interlayer galleries, as demonstrated for ionic liquid-intercalated LDH systems;^54^ and (iv) encapsulation by the hydrophobic stearic acid layer. The exceptional chemical stability (pH 1–13), mechanical durability (1050 mm abrasion), and sustained corrosion protection efficiency (99.5%) observed in this study as well as the FTIR study for the LDH–NILq composite provide an evidence that the NILq remains firmly integrated (chemically bonded) within the coating structure during prolonged exposure to aggressive environments.

### 3.10. Practical significance and scalability considerations

Beyond the excellent corrosion protection performance and mechanical durability demonstrated in this study, the practical viability of the LDH–NILq–SA coating system is further supported by several cost-related and scalability advantages. While a direct quantitative cost comparison with commercial marine coatings is challenging, as costs depend on raw material prices, production scale, and application methods, several qualitative cost benefits are evident:

(i) Low-cost precursors: the Ni–Al LDH is synthesized from inexpensive and readily available nickel salts (NiCl_2_ and NiSO_4_) with an aluminum anode serving as the Al^3+^ source, avoiding the need for costly aluminum precursors.

(ii) Green surface modifier: stearic acid, a naturally occurring and low-cost fatty acid, is employed to achieve low surface energy. This avoids the use of expensive and environmentally hazardous fluorosilanes or perfluorinated compounds commonly used in many superhydrophobic coatings.

(iii) Simple one-step electrodeposition: the coating is fabricated using a straightforward two-electrode electrodeposition system with a simple electrolyte bath. This single-step process operates at room temperature, minimizing both equipment investment and operational costs compared to multi-step or high-temperature fabrication routes.

(iv) Eco-friendliness: the system is free from toxic heavy metals (*e.g.*, zinc, copper, chromium) and fluorinated compounds, potentially reducing environmental compliance costs and enhancing the sustainability profile of the coating.

In contrast, conventional commercial marine coatings often involve multi-layer application systems (primer, intermediate tie coat, and topcoat), increasing material consumption, labor costs, and application time. Similarly, many high-performance superhydrophobic coatings reported in the literature rely on expensive fluorinated reagents or complex, multi-step fabrication processes that limit their practical scalability.

From a scalability perspective, electrodeposition is an industrially established technique already widely used in electroplating and anodizing operations, making it readily adaptable to large-scale continuous production lines. The room-temperature operation and simple bath chemistry further facilitate scale-up. However, it is acknowledged that electrodeposition is limited to conductive substrates, and further optimization is required to ensure batch-to-batch consistency for large-scale manufacturing. Nevertheless, the combination of excellent performance, low-cost precursors, a green modifier, a simple fabrication process, and environmental friendliness position the LDH–NILq–SA coating as a promising candidate for practical corrosion protection applications in marine and industrial infrastructure.

## 4. Conclusion

In this study, the optimized LDH–NILq–SA coating, prepared with 30 ppm NILq, exhibited exceptional superhydrophobic properties with a water contact angle of 166.5° and a sliding angle of 0.5°. SEM and AFM analyses revealed that the incorporation of NILq induced a systematic morphological transition from coarse nano-walls to a well-developed nano-wall architecture, with optimal surface roughness (*R*_a_ = 369.9 nm) achieved at 30 ppm NILq concentration. This hierarchical nanostructure effectively traps air pockets, minimizing solid–liquid contact and maximizing water repellency.

The coating exhibited excellent chemical stability, maintaining its superhydrophobic behavior over a wide pH range (1–13). This durability is attributed to the strong chemical interactions between NILq, stearic acid, and the LDH framework, which resist structural degradation under aggressive conditions. Furthermore, the LDH–NILq–SA coating exhibited remarkable mechanical durability, withstanding 1050 mm of abrasion under 5 kPa load -three times the abrasion resistance of the NILq-free LDH–SA coating-while maintaining superhydrophobicity. The LDH–NILq–SA coating exhibited superior thermal stability, retaining superhydrophobicity up to 200 °C compared to 150 °C for the NILq-free coating.

Electrochemical corrosion evaluation in a 0.5 M NaCl solution demonstrated that the 30 ppm LDH–NILq–SA coating achieved a protection efficiency of 99.5% from EIS measurements, significantly outperforming the NILq-free coating and bare steel. PDP analysis confirmed the suppression of both anodic and cathodic reactions. The corrosion current density followed the order: 30 ppm LDH–NILq–SA < 20 ppm LDH–NILq–SA < 10 ppm LDH–NILq–SA < LDH–SA < bare steel, confirming the progressive enhancement in corrosion resistance with increasing NILq concentration.

While the LDH–NILq–SA coating demonstrated excellent chemical (pH 1–13), mechanical (1050 mm abrasion), and thermal (up to 200 °C) stability under accelerated laboratory conditions, these evaluations do not fully encompass the complex, synergistic degradation mechanisms encountered in real marine service environments. Comprehensive testing-including long-term artificial seawater immersion, cyclic wet–dry exposure (simulating tidal zones), biofouling resistance, and combined wear-corrosion (tribocorrosion) – was beyond the scope of the present study. Nevertheless, the demonstrated regenerability of the superhydrophobic performance after thermal degradation and the exceptional structural integrity of the LDH–NILq hierarchical architecture provide strong indications of potential resilience against such harsh operational conditions. Systematic investigations addressing these aspects, including extended electrochemical monitoring and field exposure tests, are currently underway in our laboratory and will be the subject of subsequent reports.

## Conflicts of interest

There are no conflicts to declare.

## Supplementary Material

RA-016-D6RA05208H-s001

## Data Availability

All data supporting this study are included in the main article or in supplementary information (SI). Supplementary information: full spectroscopic characterization of the synthesized compounds (^1^HNMR, ^13^C NMR, FTIR, HRMS), detailed experimental materials and equipment. See DOI: https://doi.org/10.1039/d6ra05208h.
