# Supplementary material for: Engineering durable superhydrophobic layered double hydroxide coatings via incorporation of a nano-formulated quinoline-based ionic liquid for enhanced corrosion protection
Source: RSC Adv. 2026 Sep 8;16(43):53477–94. doi: 10.1039/d6ra05208h (PMC13551554; doi:10.1039/d6ra05208h)
Supplement: Supplementary file 1 [file RA-016-D6RA05208H-s001.pdf]

Supplementary Information (SI) for RSC Advances.
This journal is © The Royal Society of Chemistry 2026

Supplementary Information

Engineering durable superhydrophobic Layered double hydroxide coatings via incorporation of a nano-formulated quinoline-based ionic liquid for enhanced corrosion protection

Eman M. Saadallah [1], Ahmed R. Rabee [1], Essam M. Sharshira[1*], Mohamed Hagar [1*], Emad Matter [2], Azza M. Shaker [1], Mohamed El-shahat Mohamed [1*]

1. Chemistry Department, Faculty of Science, Alexandria University, P.O. Box 426, Alexandria, 21321, Egypt.

*E-mail: M.H., mohamed.hagar@alexu.edu.eg; M.E.M, elshahatchemist93@gmail.com; E.M.S. dressamsharshira@yahoo.com.

2. Chemistry Department, Faculty of Science, Damanhour University, Damanhour, 22511, Egypt.

**Title**	**Page number**
**Figure S1.** FTIR spectrum of compound **3**	**S3**
**Figure S2.** ^{1}H NMR spectrum of compound **5**	**S4**
**Figure S3.** Expanded ^{1}H-NMR spectrum of compound **5**	**S5**
**Figure S4.** ^{13}C NMR spectrum of compound **5**	**S6**
**Figure S5.** FTIR spectrum of compound **5**	**S7**
**Figure S6.** HRMS spectrum of compound **5**	**S8**
**3.1.** Materials and Equipment	**S9**

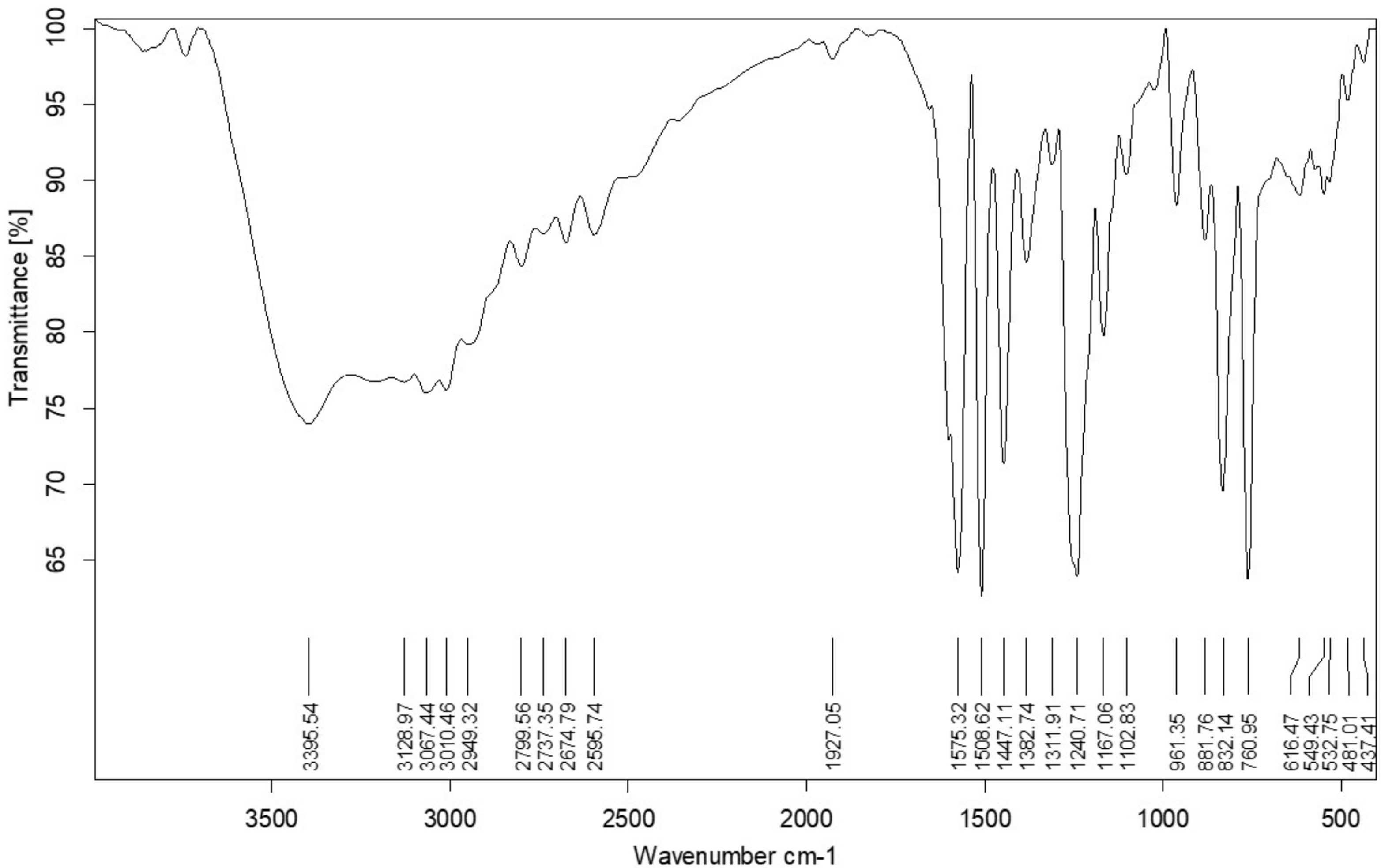

Figure S1. FTIR spectrum of compound **3**

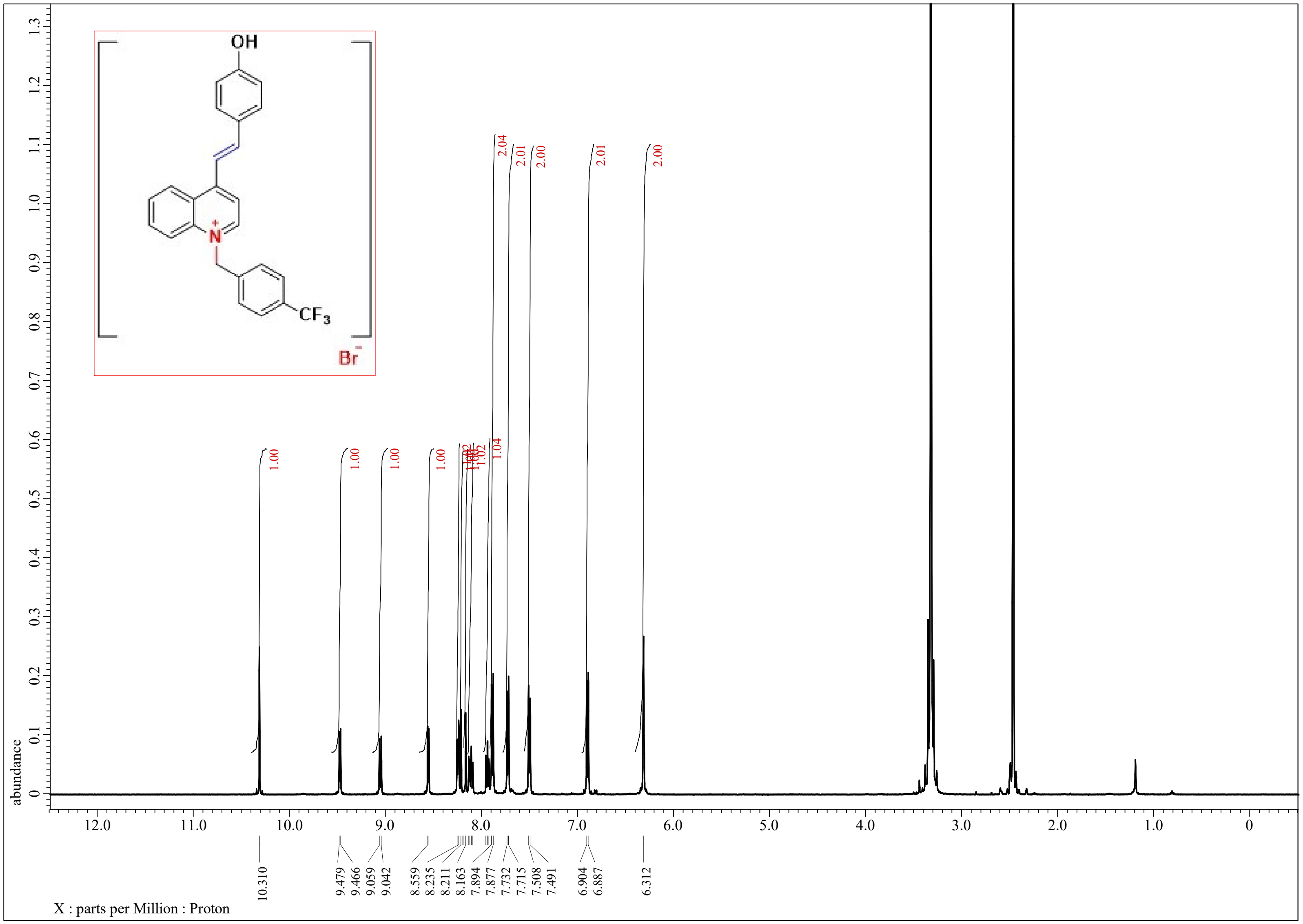

Figure S2. ^{1}H NMR spectra of compound **5**

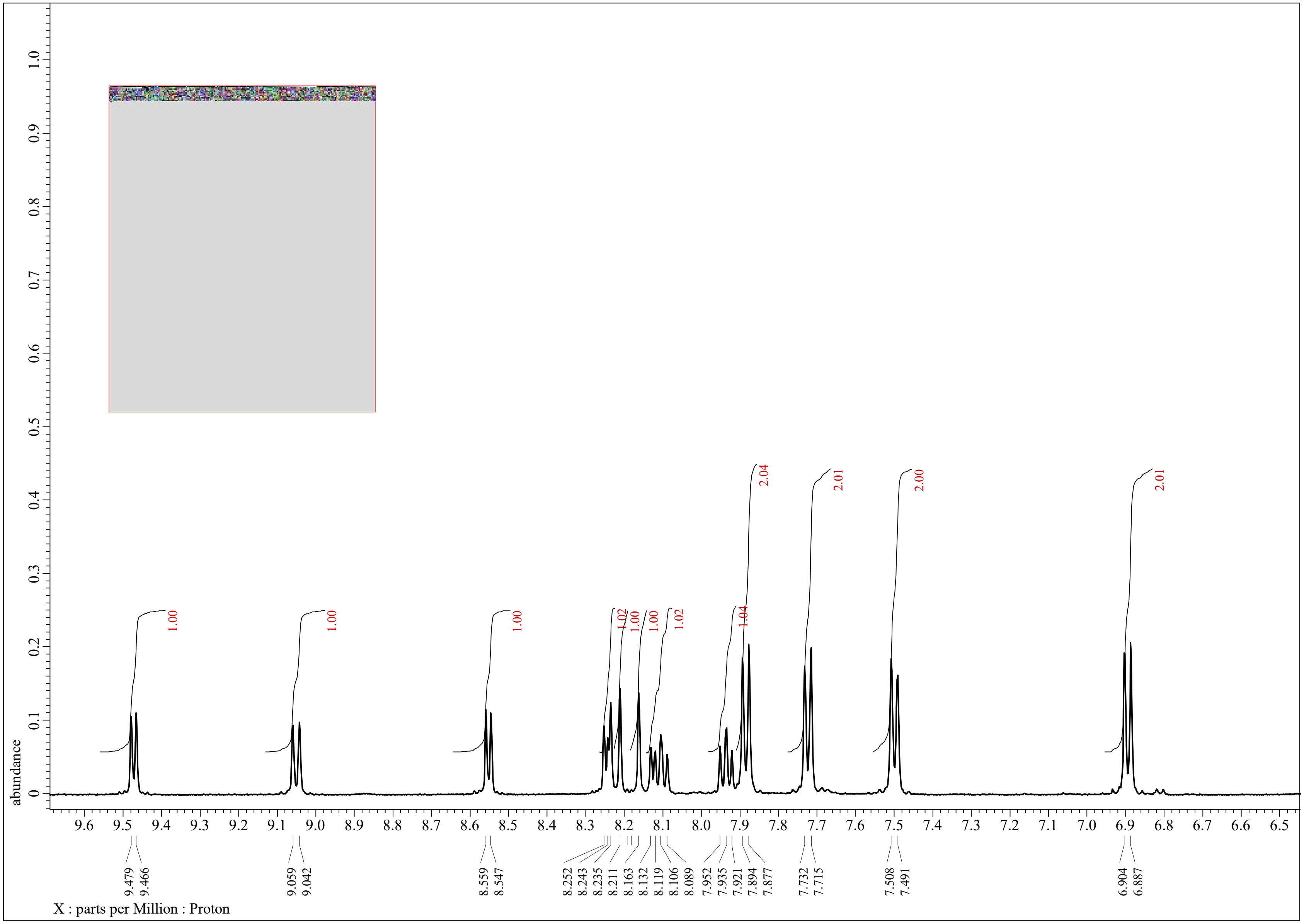

Figure S3. Expanded ^{1}H NMR spectra of compound **5**

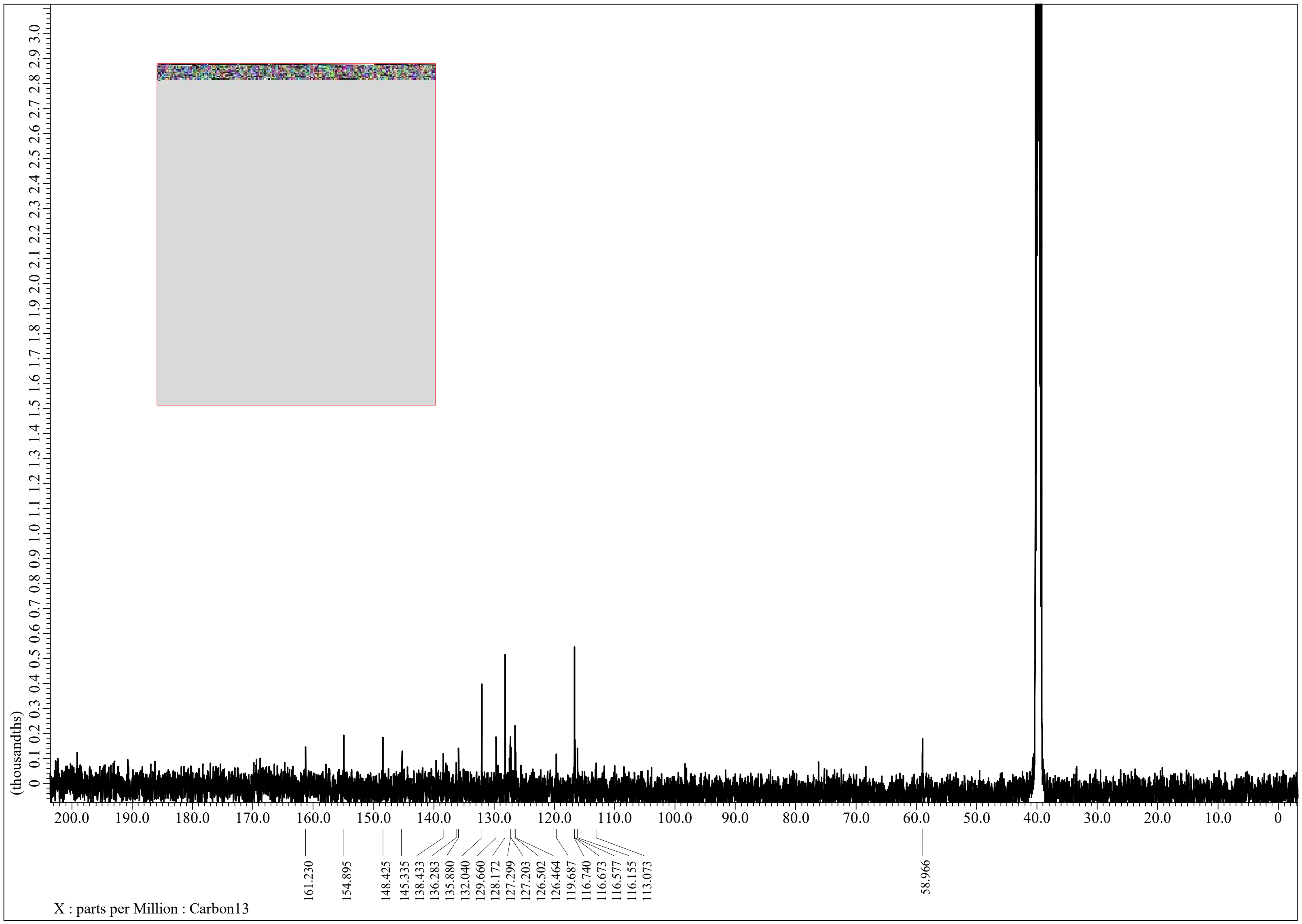

Figure S4. ^{13}C NMR (DMSO) of compound **5**

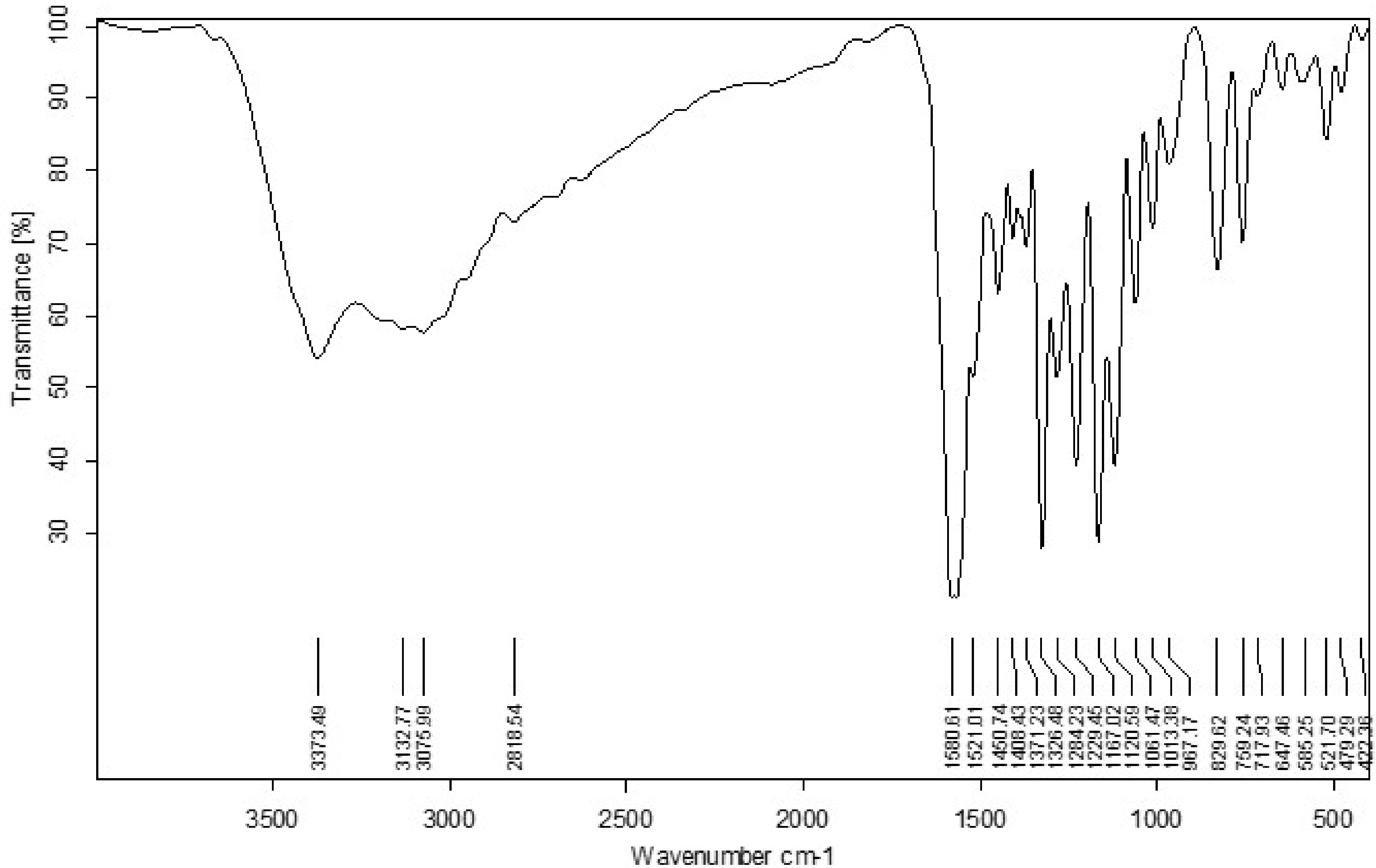

Figure S5. FTIR spectrum of compound **5**

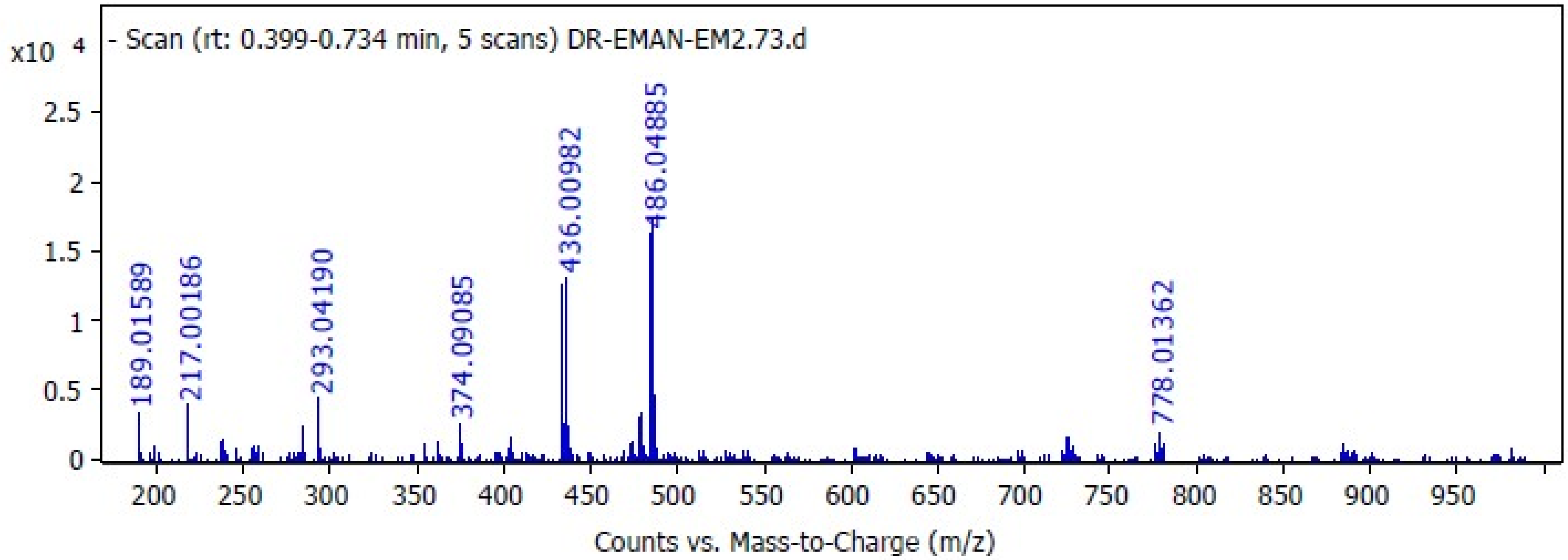

Peak List

m/z	z	Abund
189.01589		3466.01
217.00186		3970.79
293.0419		4432.49
434.01175	1	12609.12
436.00982	1	13134.1
480.02603		3462.76
484.0504	1	16349.87
485.05306	1	4453
486.04885	1	17349.5
487.05182	1	4580.14

Figure S6. HRMS spectrum of compound **5**

2. Material and Method

2.1. Materials and Equipment

The used chemicals were of analytical grade and used as received without further purification included p-Hydroxybenzaldehyde (extra pure) was purchased from Alpha Chemika (Mumbai, India). 4-Methylquinoline, chitosan, and acetonitrile were obtained from Sigma-Aldrich (St. Louis, MO, USA). Tripoly phosphate anhydrous (TPP) were obtained from Loba Chemie. 4-(Trifluoromethyl)benzyl bromide (98%) was supplied by Alfa Aesar (Ward Hill, MA, USA). Nickel chloride and nickel sulfate were procured from IsoCHEM (Cairo, Egypt). Stearic acid, sulfuric acid, sodium chloride, and DMSO were obtained from Piochem (Cairo, Egypt). Sodium hydroxide and potassium hydroxide were supplied by ADWIC (Cairo, Egypt). Ethanol 95 % was purchased from International Company for Sup. & Med. Industries (Egypt). Acetic anhydride was obtained from Techno Pharmchem (Bahadurgarh, Haryana, India). During the preparation of chemical solutions, a magnetic stirrer was operated at 500 rpm, and all solutions were synthesized via double-distilled water.

All melting points are measured on Melt-Temp apparatus Model number 1002D, 220-240 V, 200 watts, 50/60 Hz and are uncorrected. Thin layer chromatography (TLC) was performed on aluminum plates silica gel (Fluka, 60 F254 layer thickness 0.2 mm). Visualization of the TLC during monitoring of the reaction was done by UV VILBER LOURMAT 4w-365 nm or 254 nm tube. NMR spectra measured with JEOLJNM-ECA 500 MHz spectrometer, using (DMSO-d6) solvent. Chemical shifts (δ) are given in ppm relative to the signal for TMS as internal standard, and coupling constants are quoted in Hz. ^{13}C NMR spectra were recorded on a 100 MHz and 125 MHz spectrometer. The IR spectra was obtained using a Perkin-Elmer FTIR spectrum BX and Bruker tensor 37 FTIR, and wavelengths are reported in Cm^{-1}. High-resolution mass spectrometric analysis (HRMS) was carried out using an Agilent 6500 Series Q-TOF mass spectrometer equipped with an electrospray ionization (ESI) source and operated in positive ion mode. The instrument was run in full scan mode with a fragmentor voltage of 200 V, covering a mass range up to m/z 3200. Data acquisition and instrument control were performed using Agilent MassHunter software (Version B.09.00). Transmission electron microscopy (TEM) analysis was also

performed at the Electron Microscope Unit of Alexandria University to evaluate the fine structural characteristics and particles size of Nano formulations.

The surface morphology of the fabricated SHP coatings was examined using a scanning electron microscope (SEM; JEOL, model JSM-200 IT). The surface wettability was evaluated by measuring the WCA and SA using an optical contact angle goniometer (model 190-F2, Ramé-hart Instrument Co., USA). A 5 μL deionized water droplet was used for all measurements. The reported WCA and SA values represent the average of three measurements taken at different locations on each sample surface. The surface topography and roughness of the coatings were characterized by atomic force microscopy (AFM) using a Shimadzu SPM-9600 instrument.
